# Optimization of M1 macrophage targeting using a glucosylated albumin nanoplatform for ROS scavenging and mitochondrial rescue in acute kidney injury

**DOI:** 10.1186/s12951-026-04061-6

**Published:** 2026-01-29

**Authors:** Ji Yong Park, Jung Nam An, Seong Min Lee, Young Chan Ann, Eunjin Bae, Kyung Don Yoo, Yong Chul Kim, Hyeri Chae, Joo Yeon Oh, Ran Ji Yoo, Dong Ki Kim, Sung Hyun Hong, Yon Su Kim, Yun-Sang Lee, Seung Hee Yang

**Affiliations:** 1 Institute of Radiation Medicine, Seoul National University Medical Research Center, Seoul, Republic of Korea; 2 Department of Nuclear Medicine, Seoul National University Hospital, Seoul, Republic of Korea; 3 Department of Nuclear Medicine, Seoul National University College of Medicine, Seoul, Republic of Korea; 4 Cancer Research Institute, Seoul National University, Seoul, Republic of Korea; 5 Department of Internal Medicine, Hallym University Sacred Heart Hospital, Anyang, Republic of Korea; 6 Department of Internal Medicine, Gyeongsang National University College of Medicine, Jinju, Republic of Korea; 7 Department of Internal Medicine, Ulsan University Hospital, Seoul, Republic of Korea; 8 Department of Internal Medicine, Seoul National University Hospital, Seoul, Republic of Korea; 9 Department of Biomedical Sciences, College of Medicine, Seoul National University, Seoul, Republic of Korea; 10 Biomedical Research Institute, Seoul National University Hospital, Seoul, Republic of Korea; 11 Seoul National University Kidney Research Institute, Seoul, Republic of Korea; 12 Research Institute for Convergence Science, Seoul National University, Seoul, Republic of Korea; 13 Department of Molecular Medicine and Biopharmaceutical Sciences, Graduate School of Convergence Science and Technology, Seoul National University, Seoul, Republic of Korea; 14 Clichembio Inc, Seoul, Republic of Korea; 15 Department of Kidney Research Institute, Seoul National University Medical Research Center, Seoul, South Korea

**Keywords:** Acute kidney injury, Ischemia–reperfusion injury, Macrophages, Oxidative stress, Mitochondrial dysfunction, Albumin nanoplatform

## Abstract

**Background:**

Acute kidney injury (AKI) remains a major clinical challenge resulting from the intertwined processes of oxidative stress and macrophage-driven inflammation, both converging on mitochondrial dysfunction. We developed a glucosylated albumin nanoplatform (Glc^6^-AD^11^-Alb) designed to exploit glucose transporter 1-mediated uptake in inflammatory M1 macrophages while preserving the intrinsic antioxidant properties of albumin.

**Results:**

Physicochemical characterization confirmed reproducible synthesis with defined degrees of functionalization and stable physicochemical properties. *In vitro*, Glc^6^-AD^11^-Alb demonstrated selective uptake in M1 macrophages and significantly reduced intracellular reactive oxygen species, validating its dual role in immune modulation and redox regulation. Positron emission tomography imaging with Cu-64 radiolabeling revealed preferential renal accumulation in ischemia–reperfusion injury (IRI) models, supporting macrophage-targeted delivery. *In vivo*, administration of Glc^6^-AD^11^-Alb attenuated renal dysfunction, suppressed pro-inflammatory and oxidative markers, and promoted tubular regeneration in pre- and post-treatment settings. Importantly, Glc^6^-AD^11^-Alb directly protected renal tubular epithelial cells by restoring mitochondrial membrane potential under oxidative and hypoxic stress. Seahorse metabolic flux analysis further confirmed enhanced oxidative phosphorylation, reduced glycolytic dependency, and improved coupling efficiency, indicating recovery of mitochondrial bioenergetics. Transmission electron microscopy demonstrated preservation of mitochondrial ultrastructure, including intact cristae and elongated morphology, consistent with improved ATP synthesis capacity.

**Conclusions:**

Glc^6^-AD^11^-Alb acts through complementary mechanisms of macrophage-targeted immune modulation and mitochondrial protection, thereby disrupting the vicious cycle of inflammation and oxidative stress in AKI. This nanoplatform represents a clinically translatable therapeutic strategy with potential to improve outcomes in patients with ischemic kidney injury.

**Supplementary Information:**

The online version contains supplementary material available at 10.1186/s12951-026-04061-6.

## Background

Over the past century, reactive oxygen species (ROS) and reactive nitrogen species (RNS) have been extensively studied for their roles in redox signaling, cellular function, and tissue homeostasis [1]. When produced in excess, ROS and RNS cause oxidative damage to proteins, lipids, and DNA, ultimately leading to cellular dysfunction and death. This pathological redox imbalance contributes to the onset and progression of malignant tumors [2], chronic inflammatory disorders, neurodegenerative diseases, cardiovascular diseases, and ischemia–reperfusion injury (IRI) [3]. Accordingly, controlling ROS and RNS has long been recognized as a strategy to prevent excessive inflammation and limit disease progression [4]. Although both ROS and RNS contribute to oxidative stress, ROS is considered the major pathogenic mediator in AKI, and thus this study focuses specifically on ROS-driven injury mechanisms.

Acute kidney injury (AKI) represents a prototypical IRI-driven pathology in which oxidative stress and inflammation converge to drive tissue damage [5]. Infiltrating monocytes polarize into M1 macrophages at the injury site, where they amplify injury by producing ROS and pro-inflammatory cytokines [6]. These M1 macrophages also recruit neutrophils and promote tubular apoptosis, whereas alternative polarization to M2 macrophages facilitates repair through trophic factor secretion and immune resolution [7]. Studies demonstrate that macrophage depletion in early IRI reduces renal injury, whereas adoptive transfer of M1 macrophages worsens outcomes [6]. Thus, macrophages act as a double-edged sword, with M1 macrophage-derived ROS playing a particularly pathogenic role.

At the same time, proximal tubular epithelial cells (PTECs) are directly injured during ischemia–reperfusion. Oxygen restoration leads to electron leakage from mitochondrial complexes I and III, opening of the mitochondrial permeability transition pore (mPTP), cytochrome C release, and excessive mitochondrial ROS (mtROS) generation [8]. These mtROS act as cytotoxic agents—causing lipid peroxidation, mitochondrial DNA damage, and apoptosis—and as signaling molecules that further activate inflammatory cascades [9]. Thus, mitochondria-derived ROS from immune and parenchymal cells are now recognized as pivotal drivers of AKI pathogenesis [10]. Because mitochondrial dysfunction also underlies the maladaptive transition from AKI to chronic kidney disease (CKD), protecting mitochondrial integrity and redox homeostasis has emerged as a therapeutic priority [11].

Nanotechnology offers innovative solutions to these challenges by enabling targeted delivery and antioxidant therapy. Engineered nanoparticles can accumulate in the kidney and provide controlled ROS scavenging or immune modulation [12]. Noble metal nanozymes (e.g., Pt, Au, Ce) have demonstrated catalase- or superoxide dismutase–mimetic activity, effectively detoxifying ROS in injured tissues [13]. For example, platinum-based single-atom catalysts have been shown to attenuate renal IRI by scavenging ROS, reducing lipid peroxidation, and skewing macrophage polarization toward the reparative M2 phenotype [14]. Despite these advances, issues of safety, stability, and scalability remain major obstacles to clinical translation [15].

In this study, we used albumin (Alb) as an ROS trap. Alb represents a suitable nanoplatform: its free thiol group (Cys34) confers sacrificial antioxidant activity, neutralizing ROS and RNS [16]. Alb is abundant in plasma, highly biocompatible, and already widely used as a drug carrier in clinical nanomedicine [17]. Building on these properties, we engineered a glucosylated Alb nanoplatform to target inflammatory M1 macrophages via glucose recognition, while simultaneously scavenging ROS in macrophages and tubular cells. M1 macrophages upregulate glucose transporter 1 (GLUT1) and markedly increase glucose uptake to sustain their pro-inflammatory phenotype [18], providing a rationale for glucose-mediated targeting.

**Scheme 1:**
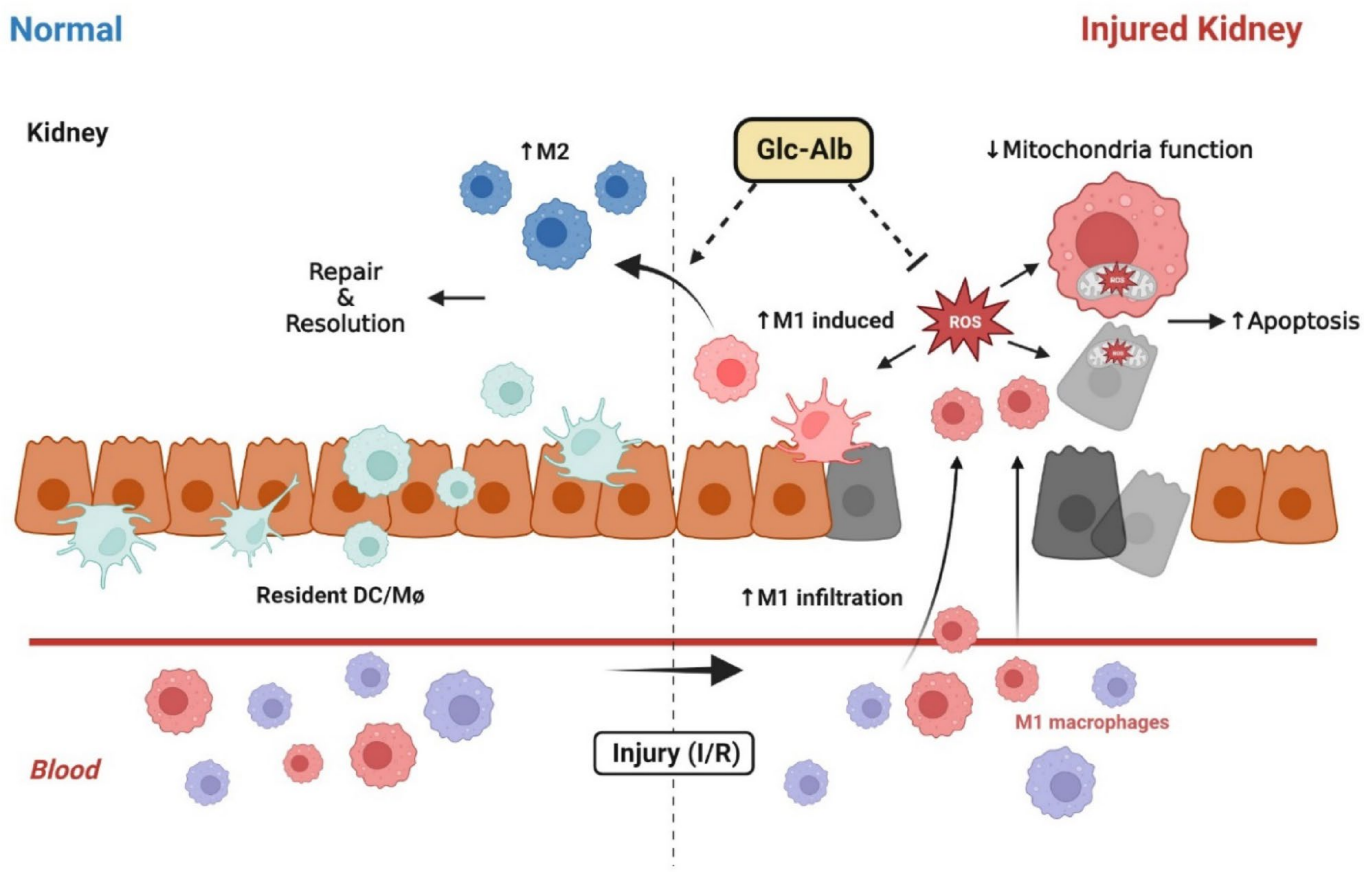
Schematic illustration of specific accumulation in renal inflammatory regions for prevention and treatment of the AKI model. The nanoplatform is selectively internalized by inflammatory cells at the lesion site, where it scavenges excessive ROS in macrophages and tubular epithelial cells, thereby mitigating oxidative stress and contributing to renal protection

We hypothesized that this strategy would restore mitochondrial function and ameliorate IRI-induced AKI by disrupting the self-perpetuating cycle of oxidative stress and inflammation. Here, we present the stepwise characterization, targeting validation, therapeutic efficacy, and mitochondrial protection achieved by our glucosylated Alb nanoplatform in *in vitro* and *in vivo* AKI models (Scheme 1).

## Methods

### Materials

6-Azido-6-deoxy-D-glucose (Glc(6)) and general reagents were purchased from Sigma-Aldrich (St. Louis, MO, USA). Exbumin (recombinant human serum albumin) was purchased from INVITRIA. Azadibenzocyclooctyne-N-hydroxysuccinimide (ADIBO-NHS), 2,2′,2″-(2-(4-(3-(3-azidopropyl)thioureido)benzyl)−1,4,7-triazonane-1,4,7-triyl)triacetic acid (NOTA-N_3_), and azido-Flamma 648 (FNR646-N_3_) were purchased from FutureChem (Seoul, Korea). Horse serum was obtained from Gibco (Madison, USA). A BCA protein assay kit was purchased from Thermo Fisher Scientific (Rockford, IL, USA). Instant thin-layer chromatography–silica gel (ITLC-SG) plates were purchased from Agilent Technologies, Inc. (Santa Clara, CA, USA). C57BL/6 (B6) mice were procured from Koatech (Seoul, Korea). Xylazine (Bayer, Canada) and Zoletil™ (Virbac, Fort Worth, TX, USA) were used for animal experiments. Microaneurysm clamps were purchased from Roboz Surgical Instrument Co. (Gaithersburg, MD, USA). For immunohistochemistry, biotin-conjugated goat anti-rabbit secondary antibodies were obtained from Vector Laboratories (Burlingame, CA, USA). 3,3′-diaminobenzidine tetrahydrochloride and Mayer’s hematoxylin were purchased from Sigma-Aldrich. RNeasy Kit, AMV-RT Taq polymerase, and primers for real-time PCR were purchased from Qiagen (GmbH, Hilden, Germany), Promega (Madison, WI, USA), and Applied Biosystems (Foster City, CA, USA), respectively. For cell culture, collagenase, DMEM/F12 medium, and collagen-coated Petri dishes were purchased from Sigma-Aldrich, Lonza, and BD Biosciences (Franklin Lakes, NJ, USA), respectively. A colorimetric MTS assay Kit was purchased from Promega. An Annexin V/propidium iodide (PI) fluorescein isothiocyanate (FITC) apoptosis kit was purchased from BD Biosciences. For JC-1 assays, hydrogen peroxide (H_2_O_2_) (H1009) and a JC-1 assay kit (M34152) were purchased from Sigma-Aldrich and Invitrogen (Carlsbad, CA, USA), respectively. For mitochondrial membrane potential measurement, MitoTracker Green (M7514) and ethidium homodimer (EthD) (L3224) were purchased from Invitrogen. The Seahorse XF Cell Mito Stress Test kit was purchased from Agilent (Santa Clara, CA, USA). A 2.5% glutaraldehyde solution and osmium tetroxide were purchased from Sigma-Aldrich and LADD Research Industries Inc. (Williston, VT, USA), respectively.

### Preparation of Glucose-Alb using a clickable Alb platform

The clickable moiety described in our previous study was employed for Alb surface functionalization, with slight modifications to the reaction conditions [19, 20]. To minimize batch-to-batch variation, we optimized the protocol by initiating the reaction with a 4-fold higher concentration and scaling up the total reaction volume 40-fold compared with prior conditions. Recombinant Alb (GMP grade) was used to reduce immunogenicity.

To prepare the Alb-ADIBO (Alb-AD) conjugate, 400 mg of recombinant human serum Alb (rHSA, Exbumin; GMP grade) was accurately weighed and placed into a 50 mL conical tube. The sample was dissolved in 10 mL of phosphate-buffered saline (PBS) with brief ultrasonication, followed by gentle stirring at room temperature to ensure complete dissolution. Separately, 100 mg of ADIBO-NHS ester was dissolved in 200 µL of dimethyl sulfoxide (DMSO) in a 1.5 mL microcentrifuge tube. After confirming complete solubilization, 36.32 mg of the ADIBO-NHS solution was dispensed into the bottom of a 50 mL conical tube. The 10 mL Alb solution was then added dropwise under mild ultrasonication. The reaction mixture was protected from light with aluminum foil and stirred at room temperature for 8 h. The molar reaction ratio of Alb to ADIBO was adjusted to 1:15. The NHS ester of ADIBO reacts with primary amine groups on Alb lysine residues, covalently attaching the ADIBO moiety to the protein. After incubation, the mixture was centrifuged at 3,000 rpm for 5 min to remove unreacted precipitates. The supernatant (10 mL) was collected and purified using an Amicon Ultra-15 centrifugal filter unit (MWCO 30 kDa) at 3,000 rpm for 10 min. The volume was restored to 10 mL with PBS, and the wash was repeated under the same conditions. The purified Alb-AD solution was carefully transferred to a 15 mL conical tube. The final volume was adjusted to 10 mL with PBS.

Next, 6-azido-6-deoxy-D-glucose (Glc–N₃) was added to the Alb-AD solution to allow strain-promoted azide–alkyne cycloaddition (SPAAC). Specifically, Alb-AD (400 mg, 40 mg/mL) was reacted with Glc-N₃ (3 mg/mL) at a molar ratio of 1:7 (Alb-AD: Glc-N₃) and incubated at room temperature for 4 h. Unreacted Glc-N₃ was removed using an Amicon Ultra-15 centrifugal filter unit (MWCO 30 kDa) at 4 °C, yielding the Glc-AD-Alb conjugate.

Ultraviolet–Visible (UV–Vis) spectroscopy was performed to confirm Glc-AD-Alb conjugation by detecting absorbance at 280 nm and 309 nm. Each sample was diluted to 2 mg/mL for measurement. The degree of functionalization (DOF) was determined by MALDI-TOF MS. For analysis, 10 µL of Alb solution was mixed with α-cyano-4-hydroxy-cinnamic acid in acetonitrile/water with 0.1% TFA (1:1, v/v). Samples (1 µL) were spotted onto MALDI plates, dried at room temperature, and analyzed in linear mode with a 25 kV accelerating voltage and 337 nm nitrogen laser. Data were collected over 125 scans (20 shots per scan), with molecular weights averaged from approximately 2,500 total laser shots. The numbers of ADIBO and glucose groups conjugated per Alb molecule were determined using UV and MALDI-TOF results, expressed as superscripts to denote functionalization levels.

The hydrodynamic diameter of Alb conjugates was measured using dynamic light scattering (DLS) on diluted samples (1–10 in PBS).

Alb modified with ADIBO groups was denoted as AD-Alb. Based on MALDI-TOF, 11 ADIBO moieties were incorporated per Alb molecule (AD^11^-Alb). Subsequent click chemistry introduced six glucose units per molecule, yielding Glc^6^-AD^11^-Alb. For clarity, constructs were referred to as AD-Alb or Glc-AD-Alb in subsequent text, as the degree of functionalization was consistent.

To prepare fluorescently labeled constructs (AD^11^-Alb-FL and Glc^6^-AD^11^-Alb-FL), N_3_-FNR648 (10 nmol/µL in DMSO) was incubated with AD^11^-Alb or Glc^6^-AD^11^-Alb (30 nmol/500 µL) at 4 ℃ for 30 min, followed by purification using centrifugal filter units.

### Target validation of AD-Alb and Glc-AD-Alb in M0 and M1 macrophages

RAW 264.7 cells were cultured in DMEM supplemented with 10% fetal bovine serum and 1% antibiotic–antimycotic solution in a 5% CO_2_ humidified incubator at 37 ℃. To polarize M1 macrophages from RAW 264.7 cells, lipopolysaccharide (LPS, 100 ng/mL) and interferon-gamma (IFN-γ, 50 ng/mL) were added to DMEM and incubated at 37 ℃ for 24 h. RAW 264.7 cells (1 × 10^5^/well) were seeded in 12-well culture plates and incubated at 37 ℃. M1 cells were polarized with LPS and IFN-γ at 37 ℃ for 24 h, beginning 24 h after seeding. To examine *in vitro* specific cellular uptake, 1 µM of Alb conjugates were added to the culture medium in each well containing M0 or M1 cells and incubated at 37 ℃ for 1 h. Samples were washed with DPBS three times before confocal laser microscopic imaging. 4’,6-diamidino-2-phenylindole (DAPI) was used for nuclear staining. Cell fluorescence imaging was performed using a confocal laser scanning microscope (STELLARIS 5, Leica, USA) with a 648 nm laser excitation, and fluorescence was collected at 671 nm and analyzed by ImageJ.

### *In vitro* ROS level test to confirm efficacy

To confirm treatment efficacy, M1 macrophage specificity, and ROS scavenging, RAW 264.7 cells were used. Cells (4 × 10^4^) were seeded in confocal dishes and incubated at 37 ℃. Pre-group cells were polarized by treatment with LPS and IFN-γ together with Alb conjugates (6.25 µM) for 24 h, initiated 24 h after seeding. The post-treatment group cells were first polarized to M1 with LPS and IFN-γ and then treated with Alb conjugates (6.25 µM) for 24 h. For ROS scavenging confocal imaging, each sample was incubated for 45 min with DCFH-DA (1 µM).

Cells were then imaged using a confocal laser scanning microscope (STELLARIS 5, Lica, USA) with a 488 nm laser excitation, and fluorescence was collected at 510 nm and analyzed by ImageJ.

### WST-1 superoxide scavenging assay

The superoxide scavenging activity of Alb and Glc-Alb was evaluated using a WST-1–based colorimetric assay [21]. Briefly, samples were prepared at concentrations of 0.25, 0.5, 1, 2, and 4 µM in assay buffer. Each sample (20 µL) was added to a 96-well plate, followed by the addition of 200 µL of WST-1 working solution and 20 uL of enzyme working solution (Dojindo, Japan). The reaction mixture was incubated for 20 min at 37 °C in the dark to allow superoxide-driven reduction of WST-1. Absorbance was measured at 450 nm using a microplate reader (BioTek Synergy). Superoxide scavenging activity was calculated according to the manufacturer’s instructions.

### Radiolabeling and stability test

The vial containing ^64^Cu was dried using N_2_ gas in a fume hood for 30 min. Subsequently, 200 µL of 1 M sodium acetate buffer (pH 5) was added to adjust the pH to 5. Then, NOTA-N_3_ (10 µL of 1 mg/mL in distilled water) was added and heated at 70 ℃ for 5 min. Thin-layer chromatography (TLC) was performed using ITLC-SG paper to determine radiolabeling efficiency (radio-TLC, 0.1 M citric acid as the mobile phase). After confirming radiolabeling efficiency, 10 µL (9 nmol, 0.5 mCi) of the radiolabeled NOTA-N_3_ solution ([^64^Cu]Cu-NOTA-N_3,_ 10 mCi/200 µL) was added to Glc^6^-AD^11^-Alb or AD^11^-Alb in PBS (500 µg/0.5 mL, 15 nmol/0.5 mL) and incubated for 30 min at room temperature. Radio-TLC was then conducted to confirm successful radiolabeling of Glc^6^-AD^11^-Alb or AD^11^-Alb (Glc^6^-AD^11^-Alb-^64^Cu or AD^11^-Alb-^64^Cu). The Rf values of the free ^64^Cu, [^64^Cu]Cu-NOTA-N_3_, and Glc^6^-AD^11^-Alb-^64^Cu or AD^11^-Alb-^64^Cu were 1.0, 0.5–0.6, and 0.0–0.1.0.1, respectively. To evaluate radiostability, AD^11^-Alb-^64^Cu, Glc^6^-AD^11^-Alb-^64^Cu, and Glc^6^-AD^11^-Alb-^64^Cu mixed with human serum were analyzed by radio-TLC at 0, 1, 4, 8, and 24 h to confirm stable conjugation of the radiolabeled agent during imaging.

### *In vivo* image-based biodistribution analysis

To obtain control images, positron emission tomography (PET) imaging was performed after intravenous injection of AD^11^-Alb-^64^Cu or Glc^6^-AD^11^-Alb-^64^Cu into mice, followed by image comparison. The injection dose for each mouse was 0.6 nmol/0.1 mL (40 µg/mouse, 2 mg/kg), based on the maximum concentration of 1,000 nM observed in the cytotoxicity experiment. This injection dose corresponded to the therapeutic concentration used in this study. Approximately 1.85 MBq of AD^11^-Alb-^64^Cu or Glc^6^-AD^11^-Alb-^64^Cu was injected intravenously into 7- to 8-week-old male wild-type mice (C57BL/6; B6) anesthetized with 2% isoflurane to confirm image-based biodistribution. PET scans were acquired at 0, 2, 4, 24, and 48 h post-injection using a preclinical PET/X-ray scanner (GENISYS4, Sofie Bioscience, California, USA). PET image processing was performed using InVivoScope software (version 2.0).

### *In vivo* PET imaging in the AKI model

To assess the targeting ability of AD^11^-Alb-^64^Cu and Glc^6^-AD^11^-Alb-^64^Cu, PET imaging was performed in an AKI model and a Sham group using the same compounds previously evaluated in normal mice. As in the normal mice experiments, equal amounts of AD^11^-Alb-^64^Cu or Glc^6^-AD^11^-Alb-^64^Cu were administered intravenously to AKI and Sham mice. PET images were acquired at 0.5, 4, 8, and 24 h post-injection. Quantitative analyses were performed to evaluate compound uptake in target tissues, particularly the kidneys, to assess their effectiveness in the AKI model versus the Sham group. Uptake patterns and intensities were compared between the two groups, providing insights into the targeting potential of AD^11^-Alb-^64^Cu and Glc^6^-AD^11^-Alb-^64^Cu in AKI. For quantitative assessment of uptake in the blood pool and kidney, the region of interest was determined using AMIDE software. Time –activity curves were generated by fitting the %ID/g (percentage of injected dose per gram) at each time point.

### Animals and establishment of the AKI model

Seven- to eight-week-old male wild-type mice (C57BL/6; B6) were housed in a specific pathogen-free animal facility. All experimental protocols were conducted in strict accordance with the Guidelines for the Care and Use of Laboratory Animals established by the National Research Council and the National Institutes of Health. Approval for the study was obtained from the Institutional Animal Care and Use Committee of the Clinical Research Institute at Seoul National University Hospital. Prior to experimentation, mice were anesthetized with a combination of xylazine (Rompun; 10 mg/kg) and Zoletil^™^ (30 mg/kg). Bilateral flank incisions were made to expose the kidney pedicles, which were clamped for 30 min using microaneurysm clamps. Mice were placed on a heating pad set at 38–39 °C in the supine position to maintain normothermia. To ensure fluid balance, prewarmed PBS (500 µL) was administered intraperitoneally. Sham-operated mice underwent identical surgical procedures, excluding renal pedicle clamping.

In the pre-treatment group, Glc^6^-AD^11^-Alb (40 µg/mouse) was intravenously administered 1 h prior to induction of bilateral IRI (bIRI), with kidney excision performed 48 h post-injury. In the post-treatment group, the same dose of Glc^6^-AD^11^-Alb was administered 3 h after ischemia induction.

### Assessment of kidney function

At 48 h post-injury, blood urea nitrogen (BUN, mg/dL) and creatinine (Cr, mg/dL) concentrations were measured using an autoanalyzer (Hitachi Chemical Industries, Ltd., Osaka, Japan) via the modified Jaffe reaction method.

### Histological analysis

Paraffin-embedded kidney Sect.(4 μm) were stained with periodic acid–Schiff reagent and examined under a light microscope. For immunohistochemistry, 4 μm sections were deparaffinized and rehydrated with xylene and ethanol. Endogenous peroxidase activity was blocked using 3% H_2_O_2_. Sections were then stained with antibodies against NGAL, E-cadherin, Cytochrome C, superoxide dismutase 1 (SOD-1), ICAM-1, F4/80, iNOS, p21, 8-OHdG, and proliferating cell nuclear antigen (PCNA), followed by incubation with biotin-conjugated goat anti-rabbit secondary antibodies. Immunohistochemical signals were detected using streptavidin and 3,3′-diaminobenzidine tetrahydrochloride. Sections were counterstained with Mayer’s hematoxylin and evaluated under a Leica inverted microscope (Leica Camera, Wetzlar, Germany). For quantitative analysis, 5–8 randomly selected fields (100× magnification) were analyzed using LAS-4000 software (Leica Camera) to determine the percentage of positive areas. Renal histological damage was assessed in a blinded fashion and scored on a semi-quantitative scale of 0–4, as previously described [22]. Ten cortical fields per mouse were examined, and the percentage of affected area (tubular dilation, epithelial necrosis, and intratubular cast formation) was recorded, with scores ranging from 0 (no damage) to 4 (> 75% damage). All analyses were reviewed and confirmed by a renal pathologist blinded to experimental groups.

### Quantitative reverse transcription-polymerase chain reaction (qRT-PCR)

In brief, 1 µg of total RNA was isolated from kidney samples using the RNeasy Kit, followed by reverse transcription with oligo(dT) primers and AMV-RT Taq polymerase. Real-time PCR analysis was then performed using Assay-on-Demand TaqMan probes and primers targeting NGAL, E-cadherin, cytochrome C, SOD-1, p21, MCP-1, iNOS, arginase, IL-18, and glyceraldehyde 3-phosphate dehydrogenase (GAPDH) on an ABI PRISM 7500 Sequence Detection System (Applied Biosystems). mRNA levels were determined using the comparative Ct method (2-^ΔΔCt^), with normalization to GAPDH expression.

### Cell culture and the establishment of oxidative stress model

In accordance with the guidelines approved by the Institutional Review Board of Seoul National University Hospital (IRB no. 1404-117-515), human proximal tubular epithelial cells (hPTECs) were isolated from normal tissue obtained from patients undergoing nephrectomy for renal cell carcinoma. After dissection of the cortex, tissues were finely minced and digested in Hank’s balanced salt solution containing 3 mg/mL collagenase at 37 °C for 1 h. Cortical tubular cells were then isolated through sequential sieving (150, 120, 70, and 40 μm) with PBS washes and centrifugation at 500 g for 5 min. The cells were cultured in DMEM/F12 medium on collagen-coated Petri dishes until colony formation was established. Cells from passages 2–3 were utilized in this study. After 3 days, cells were detached with 3 mM EDTA and minimal trypsin, then seeded at a density of 2 × 10^5^ cells per well on 6-well chamber slides. Following 24 h of serum starvation, cells were washed twice with PBS. Experiments were conducted with cells at passage numbers below 15, and morphology was routinely monitored by microscopy. To establish an oxidative stress model, hPTECs were treated with H_2_O_2_ (1 mM) alone or co-treated with Glc^6^-AD^11^-Alb (1 µM) for 24 h.

### MTS assay

Cell proliferation was assessed using colorimetric MTS assay kits according to the manufacturer’s instructions. Briefly, MTS [3-(4,5-dimethylthiazol-2-yl)−5-(3-carboxymethoxyphenyl)−2-(4-sulfophenyl)−2 H-tetrazolium] and phenazine methosulfate (PMS) were combined at a 20:1 ratio immediately before addition to the cells, with protection from light. The MTS/PMS solution (20 µL) was added to each well and incubated at 37 °C in a humidified 5% CO_2_ atmosphere for 100 min. Absorbance was measured at 490 nm using a microplate reader (SpectraMax 250; Molecular Devices, Sunnyvale, CA, USA).

### Annexin V/PI staining assay

Apoptosis and necrosis were quantified utilizing an Annexin V/PI FITC apoptosis kit. hPTECs were suspended in 100 µL of binding buffer, after which 5 µL of FITC-conjugated Annexin V (10 mg/mL) and 10 µL of PI (50 mg/mL) were added. Cells were incubated for 15 min at room temperature in the dark, following the manufacturer’s instructions. Data acquisition and analysis were performed using BD FACSDiva^™^ software (version 8.0; BD Biosciences, Becton, Dickinson and Company, CA, USA).

### JC-1 assay

hPTECs were treated with H_2_O_2_ (1 mM) for 40 min to induce oxidative stress-related renal damage. For the hypoxia experiment, hPTECs were exposed to a hypoxia chamber (1% O_2_) for 24 h to assess the antioxidative properties of Glc^6^-AD^11^-Alb. Briefly, the JC-1 assay kit was employed to evaluate mitochondrial membrane potential according to the manufacturer’s instructions. Following the 40-minute incubation, JC-1 (10 µL of 200 µM) was added and further incubated at 37 ℃ in a 5% CO_2_ environment for 30 min. JC-1 was detected at 488 nm and 633 nm utilizing High-Content Screening (HCS; PerkinElmer, Waltham, Massachusetts). DAPI was used for counterstaining, after which images were captured and analyzed.

### Mitochondrial membrane potential measurement in primary tubular cells

The assessment of potential changes in the mitochondrial membrane under oxidative stress was conducted using the MitoTracker kit. Human hPTECs were labeled with 100 nM MitoTracker Green and 0.2 µL per sample of EthD. Following staining, the cells were incubated for 15 min. Fluorescence was then measured at 488 nm (MitoTracker Green) to assess mitochondrial membrane potential and at 617 nm (EthD) to evaluate live cells. DAPI staining was used for counterstaining, and images were captured and analyzed using High-Content Screening (HCS; PerkinElmer, Waltham, Massachusetts).

### Seahorse assay

The Seahorse XF Cell Mito Stress Test was utilized to measure the oxygen consumption rate (OCR) in response to 1 mM H_2_O_2_ and varying concentrations of Glc^6^-AD^11^-Alb (0.5 and 1 µM). Kidney cells were plated on the Seahorse XF96 Cell Culture Microplate, and measurements were conducted according to the manufacturer’s instructions. Non-mitochondrial respiration was subtracted to determine the basal cellular OCR. Oligomycin (2 µM) was then added to inhibit complex V of the electron transport chain, and ATP-linked respiration was calculated by subtracting the post-oligomycin rate from the baseline OCR. Proton leak respiration was determined by subtracting the non-mitochondrial respiration from the oligomycin rate. To maximize electron transport chain activity by collapsing the inner membrane gradient, carbonyl cyanide-p-trifluormethoxyphenyl-hydrazon (FCCP; 1.5 µM) was added, and maximal respiratory capacity was calculated by subtracting the non-mitochondrial respiration from the FCCP rate. Finally, antimycin A (complex III inhibitor) and rotenone (complex I inhibitor) (Rotenone/AA, 0.5 µM) were introduced to measure non-mitochondrial respiration.

### Measurement of ATP levels

Cellular ATP levels were measured using an ATP detection kit (Abcam; ab113849) according to the manufacturer’s instructions. Luminescence was measured using a microplate reader (Promega; Gloma× 96 microplate luminometer).

### Electron microscopy (EM)

Kidney sections from Sham, bIRI, and bIRI + Glc^6^-AD^11^-Alb treated groups were fixed with 2.5% glutaraldehyde solution, post-fixed in osmium tetroxide, and embedded. For EM, tissues were stained with lead citrate and uranyl acetate. Images were captured using a JEM-1400 electron microscope (JEOL Ltd., Akishima, Tokyo, Japan) at magnifications of 5000×, 30,000×, and 50,000×, focusing on the mitochondria of renal tubules. Mitochondrial morphology and cristae volume density were examined and compared across treatment groups.

### Statistical analysis

Data are expressed as the mean ± standard error of the mean (SEM) or the median (interquartile range), based on the results of the Shapiro–Wilk normality test. Student’s t-test or the Mann–Whitney U test was used as appropriate. Statistical analyses were performed using SPSS version 22 (IBM, USA) and GraphPad Prism version 10.0 (GraphPad Software, Inc., San Diego, CA, USA). Statistical significance was set at *P* < 0.05.

## Results and discussion

### Construction and characterization

**Fig. 1:**
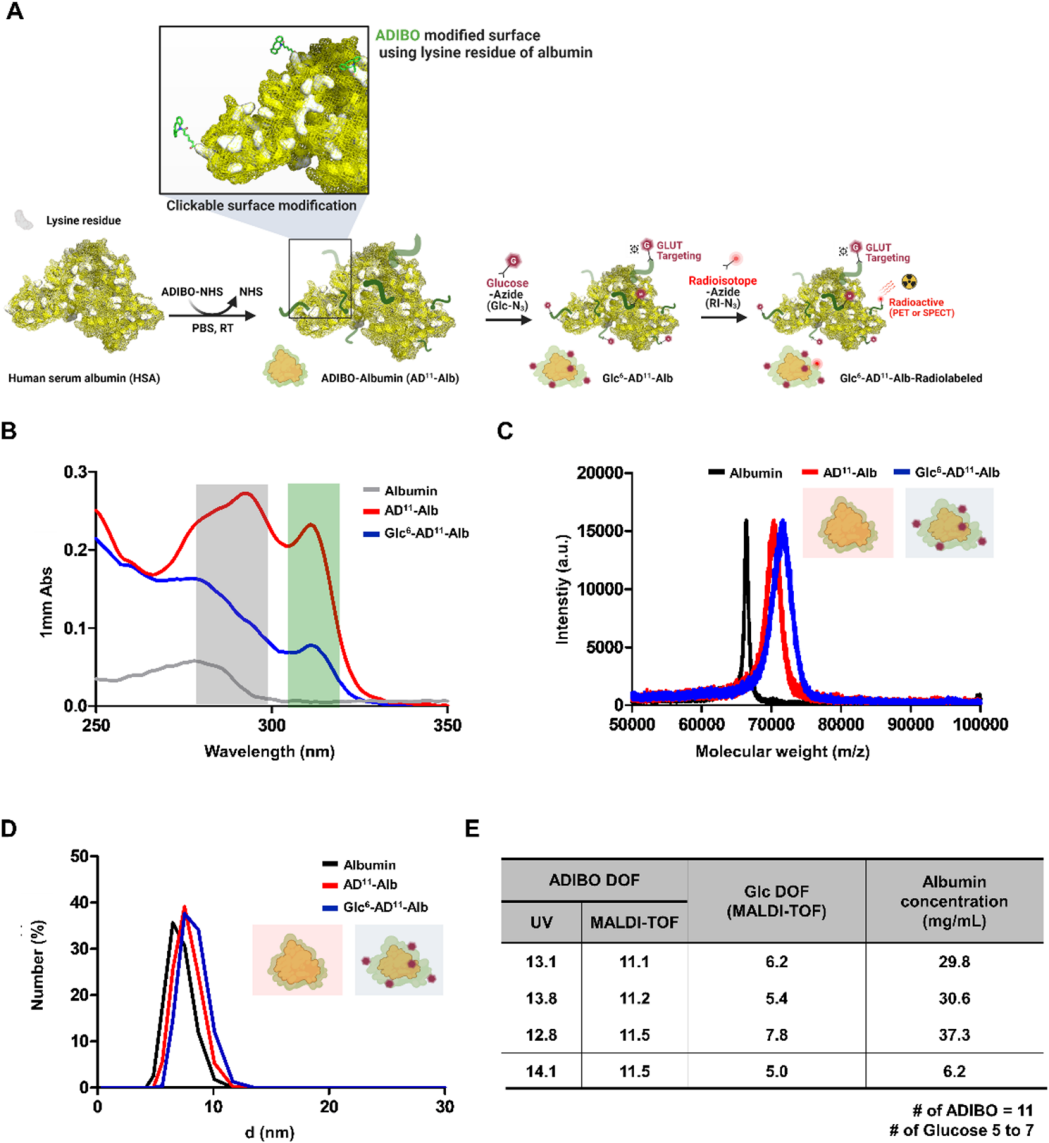
Characterization of glucosylated albumin nano-therapeutic agent. **(A)** Schematic representation of the assembly steps of the glucosylated albumin nanoplatform. **(B)** Quantification of UV-based Glc-Alb and AD-Alb. The gray box represents the characteristic peak changes of albumin, whereas the green box indicates the peak changes at 312 nm resulting from ADIBO. As the reaction with glucose-azide proceeds, the ADIBO peak decreases. **(C)** Molecular weight measurements at each assembly step using MALDI-TOF analysis. **(D)** Size measurements of nanoparticles at each assembly step using Dynamic Light Scattering (DLS). In panels **B**-**D**, black or gray represents albumin, red represents ADIBO-modified clickable albumin (AD-Alb), and blue represents AD-Alb modified with glucose (Glc-AD-Alb). **(E)** Quantification of ADIBO and glucose functionalization (DOF) of albumin determined by UV and MALDI-TOF analyses

The stepwise synthesis of the glucosylated Alb nanoplatform is illustrated in Fig. 1a. In summary, AD^11^-Alb, with 11 DOFs, was prepared by controlling the reaction between ADIBO-NHS and lysine residues on the Alb surface at a 1:15 molar ratio. The same 1:15 reaction was performed in a scale-up manner, yielding over 95% when carried out at two different concentrations (30 mg/mL and 40 mg/mL) (Fig. 1e). The properties of each product were confirmed using UV–Vis spectroscopy and MALDI-TOF mass spectrometry. UV–Vis spectroscopy validated the incorporation of ADIBO and glucose moieties into Alb. As shown in Fig. 1b, the peak at 280 nm corresponded to Alb, whereas the characteristic peak at 309 nm indicated the presence of the ADIBO functional group. During glucose-azide conjugation, a progressive decrease in the 309 nm signal was observed, consistent with glucose substitution. DOF values were calculated from UV absorbance using the equation established in our previous work [20] (Fig. 1e). MALDI-TOF mass spectrometry provided quantitative confirmation of molecular composition (Fig. 1c). By analyzing mass shifts, the number of ADIBO residues conjugated to Alb and the number of glucose molecules subsequently introduced into AD-Alb were determined. Across multiple independent syntheses, AD-Alb consistently exhibited 11 ADIBO residues, whereas glucose substitution ranged from five to seven moieties. For subsequent experiments, the average value was used, and the formulations were denoted AD^11^-Alb and Glc^6^-AD^11^-Alb, respectively (Fig. 1e). Similar reactions and yields were observed for mannose in previous investigations [20]. Additionally, DLS confirmed the absence of aggregation or degradation in AD^11^-Alb, Glc^6^-AD^11^-Alb, and unreacted Alb. The number-mean values indicated diameters of 6.944 ± 1.07 nm for Alb, 7.595 ± 1.10 nm for AD^11^-Alb, 8.144 ± 1.14 nm for Glc^6^-AD^11^-Alb, showing no significant size changes for AD^11^-Alb and Glc^6^-AD^11^-Alb, consistent with previous reports (Fig. 1d). This method, particularly for precise evaluation of the number of surface-bound molecules per particle, addressed limitations in earlier nanoparticle studies and established a robust quantitative approach [23–25]. Such precision is critical in nanomedicine for defining accurate nanoparticle specifications.

### Glucosylation-dependent targeting of M1 macrophages

**Fig. 2:**
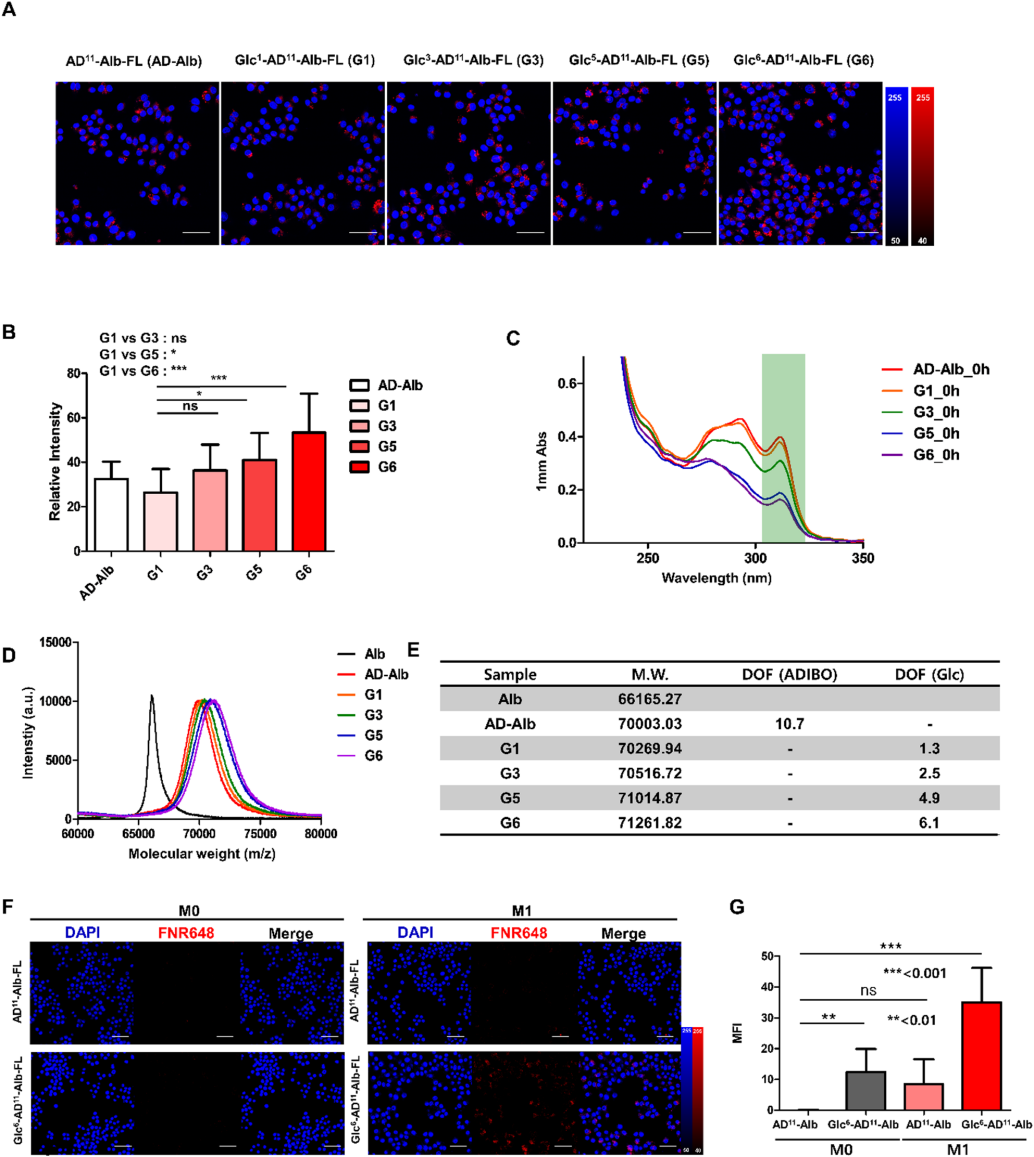
Optimization of M1 macrophage targeting via controlled glucosylation of albumin nanoplatforms. **(A)** Fluorescence experiments assessing M1 targeting ability based on the number of glucose moieties introduced into AD-Alb. Experimental groups were designated as G1, G3, G5, and G6 according to the number of glucose moieties. **(B)** Quantification of fluorescence intensity in the cell experiments (*: *P* < 0.05, ***: *P* < 0.001). **(C)** Quantification of UV-based Glc-Alb and AD-Alb. As the reaction with glucose-azide proceeds, the ADIBO peak decreases. **(D)** Molecular weight measurements at each assembly step using MALDI-TOF analysis. **(E)** Table of molecular weight results for each albumin therapeutic agent obtained by MALDI-TOF. **(F)** Cell targeting experiments using polarized macrophages (M0 and M1). Red indicates the fluorescent signal from each albumin nanoplatform, and blue represents DAPI staining. **(G)** Mean fluorescence intensity (MFI) values of the fluorescent signals in the cellular images (**: *P* < 0.01, ***: *P* < 0.001). A white scale bar indicates 50 μm

To determine whether glucose functionalization could improve the M1-targeting ability of the Alb nanoplatform, we systematically varied the number of glucose moieties conjugated to AD^11^-Alb. Fluorescence microscopy revealed that Glc-Alb nanoplatforms with increased glucosylation exhibited stronger binding and uptake in polarized M1 macrophages compared to non-modified AD^11^-Alb (Fig. 2a). Quantification of fluorescence intensities demonstrated a stepwise enhancement in cellular uptake with increasing glucose numbers, with Glc^6^-AD^11^-Alb showing the highest signal among the tested groups (G1, G3, G5, and G6) (Fig. 2b). Statistical analysis confirmed significant differences between Glc^6^-AD^11^-Alb and the lower-glucose formulations (*P* < 0.01).

UV–Vis and MALDI-TOF analyses verified the successful stepwise conjugation of glucose to Alb, with clear decreases in the ADIBO absorption peak at 312 nm and corresponding mass increases consistent with the number of glucose substitutions (Fig. 2c–e). These physicochemical characterizations ensured that differences in targeting ability were attributable to glucose number rather than variations in nanoparticle size or aggregation.

The improved targeting observed in Glc^6^-AD^11^-Alb can be explained by the overexpression of GLUT1, a metabolic marker of M1 macrophages. GLUT1-mediated glucose metabolism has been shown to drive the pro-inflammatory phenotype of M1 macrophages [18]. Previous studies demonstrated that glucose conjugation at the C6 position of polymeric micelles maximized GLUT1-mediated uptake [26]. This targeting behavior is consistent with our recent findings showing that C6-linked glucosylated (Glc(6)) albumin exhibits GLUT1-associated uptake in vivo. In that study, PET imaging and spatial transcriptomics demonstrated that Glc(6)-Alb preferentially accumulated in Slc2a1 (GLUT1)-enriched inflammatory regions, whereas non-glucosylated or differently positioned glucosylated albumin did not. Supplementary analyses further indicated that M1 macrophages, which upregulate GLUT1 under inflammatory activation, internalized Glc-functionalized albumin more efficiently than other macrophage phenotypes. These mechanistic insights support that the enhanced M1 selectivity observed for Glc^6^-AD^11^-Alb in the present study is attributable to GLUT1-mediated recognition of the C6-glucose motif on the albumin nanoplatform [27].

Inspired by this, we employed a C6-azide glucose derivative to functionalize Alb. Furthermore, ligand density has been reported as a key determinant of nanoparticle–GLUT1 interactions, with intermediate surface densities achieving deeper tissue penetration and more efficient cellular uptake compared to high-density formulations [28, 29]. In line with these findings, our results highlight the importance of tuning glucose number to achieve optimal macrophage targeting.

To evaluate the M1 targeting ability of the optimized Glc^6^-AD^11^-Alb, we utilized the Raw 264.7 cell line and polarized the cells into M0 and M1 states. Subsequently, we treated them with AD^11^-Alb-FL or Glc^6^-AD^11^-Alb-FL. In the uptake confirmation experiment using confocal microscopy, we observed high uptake of Glc^6^-AD^11^-Alb-FL in M0 and M1 states. Particularly, when the cells were polarized into the M1 state, the highest uptake was observed (Fig. 2f). Fluorescence quantification confirmed that Glc^6^-AD^11^-Alb-FL showed significantly higher uptake in M1 macrophages (Fig. 2g, Table S1). Specifically, uptake was over 4-fold higher in M1 macrophages treated with Glc^6^-AD^11^-Alb-FL compared to those treated with AD11-Alb-FL, and over 3-fold higher compared to M0 macrophages treated with Glc^6^-AD^11^-Alb-FL. These results demonstrate the highly selective uptake of Glc^6^-AD^11^-Alb in M1 macrophages.

### *In vitro* targeting and ROS scavenging effect

**Fig. 3:**
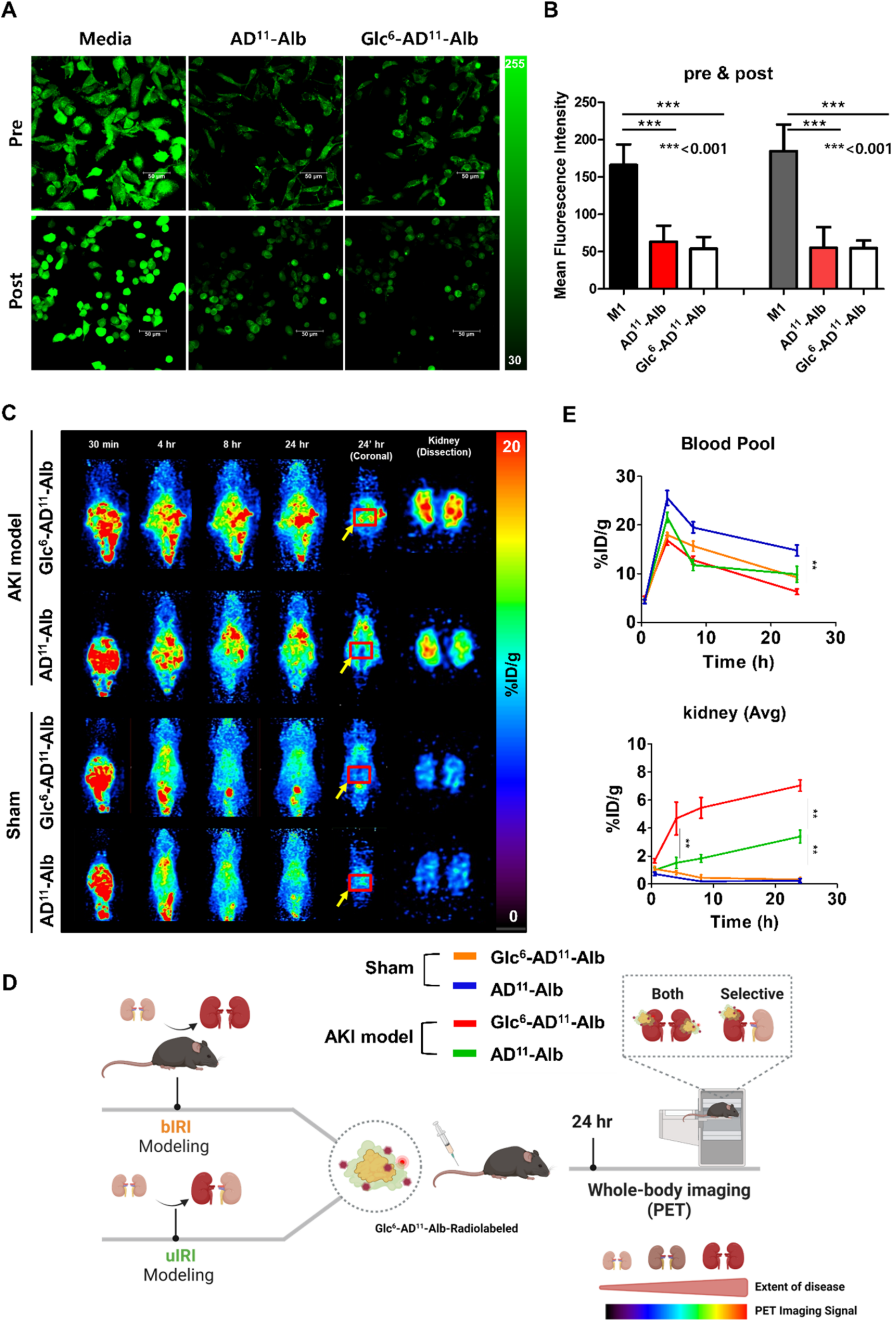
*In vitro* and *in vivo* efficacy of the glucosylated albumin nano-therapeutic agent. **(A)***In vitro* ROS level test confirming efficacy. The green fluorescent signal correlates with intracellular ROS levels. All experimental groups were polarized into M1 macrophages, with the group treated with the albumin nano-agent before polarization labeled as “Pre” and the group treated after polarization labeled as “Post.” **(B)** Quantification of confocal laser scanning microscope images. The AD-Alb group is represented by a red bar graph, and the glucosylated group is represented by a white bar graph. **(C)** Representative PET images of the AKI model and Sham group. *In vivo* biodistribution analysis confirmed targeting ability. The red box and yellow arrow indicate the kidney region. After 24 h of imaging, the kidneys were extracted for PET confirmation. **(D)** Schematic flow of the targeting confirmation experiment using bIRI and uIRI models. Disease severity and targeting ability were evaluated through PET kidney signals. **(E)** Time–activity curves of the blood pool and kidney (**: *P* < 0.01, ***: *P* < 0.001)

Next, we evaluated the ROS scavenging function of Alb at the cellular level. As mentioned earlier, ROS imbalance, or oxidative stress, is detrimental to renal tubules and ultimately leads to AKI. Therefore, we examined whether Glc^6^-AD^11^-Alb could scavenge ROS in two conditions: polarization of macrophages from M0 to M1, and polarization of already established M1 macrophages. In pre- and post-conditioning, we observed a significant reduction in ROS levels (Fig. 3a). Quantification revealed that ROS levels in Glc^6^-AD^11^-Alb– or AD^11^-Alb–treated M1 macrophages were reduced to approximately one-third of those in untreated M1 macrophages (Fig. 3b). This result was obtained at a concentration of 1 µM of Alb. These findings confirm the significant ROS scavenging effect of Alb nanoparticles, demonstrating their potent ability to reduce ROS levels in M1 macrophages. Importantly, the intrinsic scavenging function of Alb was preserved regardless of glucose modification. This finding was further supported by a WST-1 superoxide scavenging assay, which confirmed that Glc^6^-AD^11^-Alb and AD^11^-Alb retained antioxidant activity comparable to native albumin across a range of concentrations (Fig. S1). Thus, our nanoparticles hold potential for the prevention or treatment of AKI by reducing ROS via the inherent scavenging capability of Alb, independent of glucosylation.

### PET imaging-based targeting study using Cu-64-labeled Alb nanoplatform

From a translational perspective, optimizing the degree of glucosylation is critical to balance M1 macrophage targeting efficiency with systemic distribution and pharmacokinetics. To confirm the actual *in vivo* distribution of AD^11^-Alb and Glc^6^-AD^11^-Alb, we performed longitudinal PET imaging analysis. Initially, we obtained images using healthy B6 mice. For this purpose, we required a labeling agent composed of a chelator capable of binding the radionuclide and an N_3_ moiety for click chemistry with ADIBO on the Alb surface. Following previously described approaches, we used N_3_-NOTA to label the radionuclide and introduced it into the Alb platform using a pre-labeling and post-conjugation method [19, 20]. This separation of radionuclide labeling and Alb conjugation preserved physiochemical compatibility, accounting for the pH sensitivity of the biomaterial. Radiolabeling of Free ^64^Cu, [^64^Cu]Cu-NOTA-N_3_, Glc^6^-AD^11^-Alb-^64^Cu, and AD^11^-Alb-^64^Cu achieved a labeling efficiency of over 99% (Fig. S2a). In the stability test, conducted under the same time points as the imaging protocol, labeling stability remained above 94% for AD^11^-Alb-^64^Cu and Glc^6^-AD^11^-Alb-^64^Cu in serum up to 24 h (Fig. S2b).

In the PET imaging study with healthy animals, AD^11^-Alb-^64^Cu and Glc^6^-AD^11^-Alb-^64^Cu showed similar distribution (Fig. S3). The circulation time and the half-life of AD^11^ were consistent with previous reports (Table S2) [20]. However, Glc^6^-AD^11^-Alb-^64^Cu was excreted via the intestines starting from 4 h, suggesting hepatic uptake followed by intestinal excretion. In contrast, AD^11^-Alb-^64^Cu remained in the liver up to 24 h, consistent with uptake by Kupffer cells rather than hepatocytes. Neither formulation showed significant renal uptake or retention, as Alb is not excreted by the kidneys. The urinary signal represented metabolites processed by the liver and excreted thereafter.

To test the hypothesis of targeting inflammatory macrophage infiltration in the renal region in an IRI model of AKI, we used bIRI and unilateral IRI (uIRI) models (Fig. 3d). The bIRI model mimics acute kidney inflammation, whereas the uIRI model represents a chronic environment. We assessed the renal uptake of AD^11^-Alb-^64^Cu and Glc^6^-AD^11^-Alb-^64^Cu during the first 24 h post-injury using PET imaging.

In the bIRI model, Sham and AKI groups showed rapid uptake in the abdominal and wound areas after intravenous injection of AD^11^-Alb-^64^Cu and Glc^6^-AD^11^-Alb-^64^Cu (Fig. 3c). From 4 h onward, rapid redistribution into the bloodstream was observed (Fig. 3e). In the Sham group, both formulations exhibited prolonged circulation, differing from the AKI group. In the AKI model, significant renal retention was observed. Particularly, Glc^6^-AD^11^-Alb-^64^Cu showed uptake of 7.03 ± 0.32%ID/g in both kidneys, more than double the uptake of Alb (3.39 ± 0.36%ID/g) at 24 h post-injection. In the Sham group, Glc^6^-AD^11^-Alb-^64^Cu uptake was 0.32 ± 0.11%ID/g, and Alb uptake was 0.24 ± 0.12%ID/g, indicating minimal accumulation. Dissection imaging confirmed significant renal uptake of Glc^6^-AD^11^-Alb-^64^Cu. The slight Sham signal was likely attributed to blood pooling, as circulation was higher in Sham animals. All videos and sectional images over time are provided in Figure S4.

We also evaluated targeting in the uIRI model. Although uIRI mimics CKD, the influx of inflammatory cells and inflammatory microenvironment in the injured kidney during the first 24 h are similar to bIRI.

We administered Glc^6^-AD^11^-Alb-^64^Cu intravenously and obtained images at 0, 2, 4, 8, and 24 h (Fig. S5). Sectional analysis confirmed that Glc^6^-AD^11^-Alb-^64^Cu was not taken up in the healthy kidney but accumulated strongly in the injured kidney. Dissection imaging confirmed a strong PET signal in the renal parenchyma, visualizing Glc^6^-AD^11^-Alb-^64^Cu accumulation in the diseased kidney. These findings demonstrate its ability to target the early inflammatory environment in real time *in vivo*. We plan to evaluate its therapeutic effect in an AKI model through histological, genetic, and hematological analyses.

### Glc^6^-AD^11^-Alb attenuates inflammation and promotes tubular regeneration

**Fig. 4:**
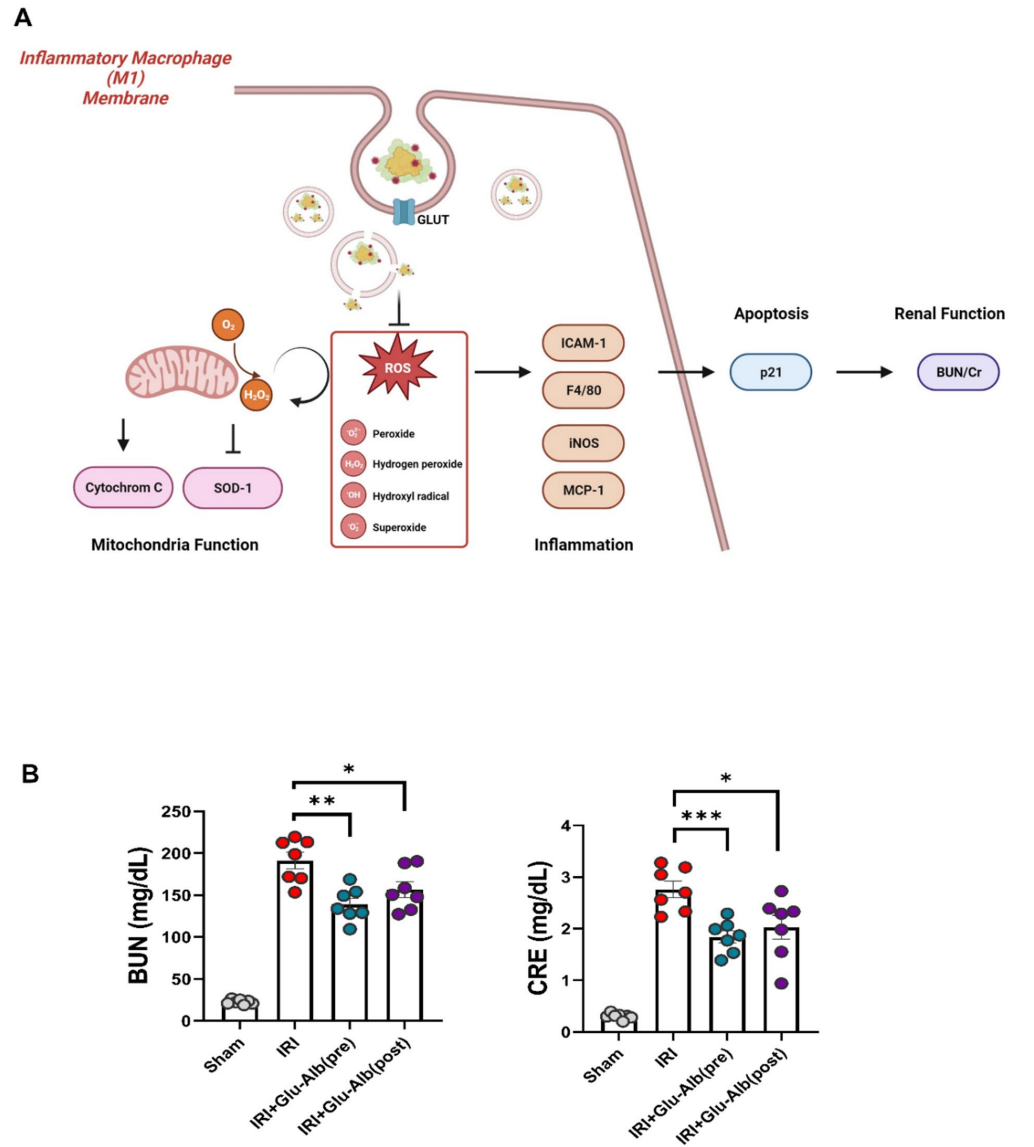

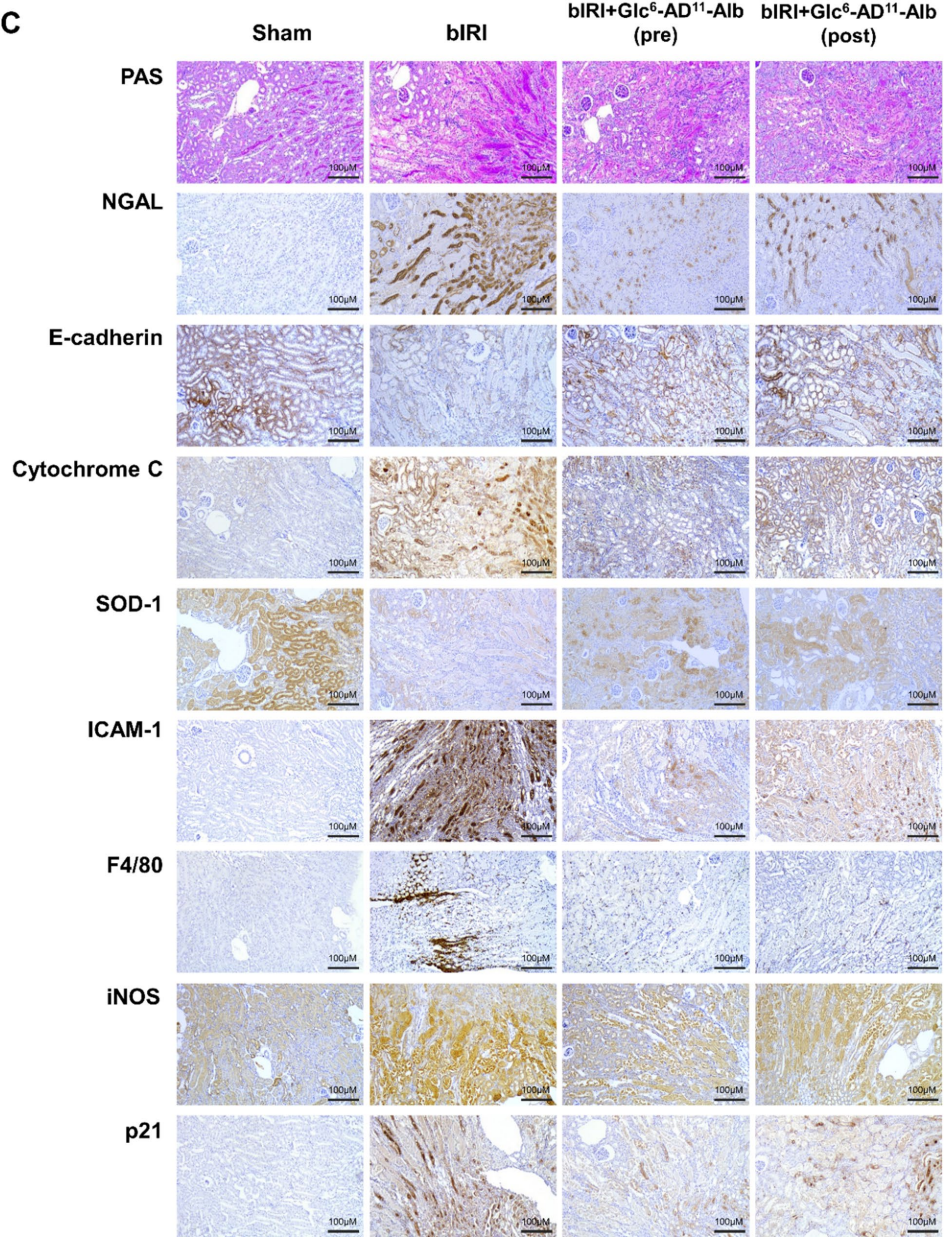

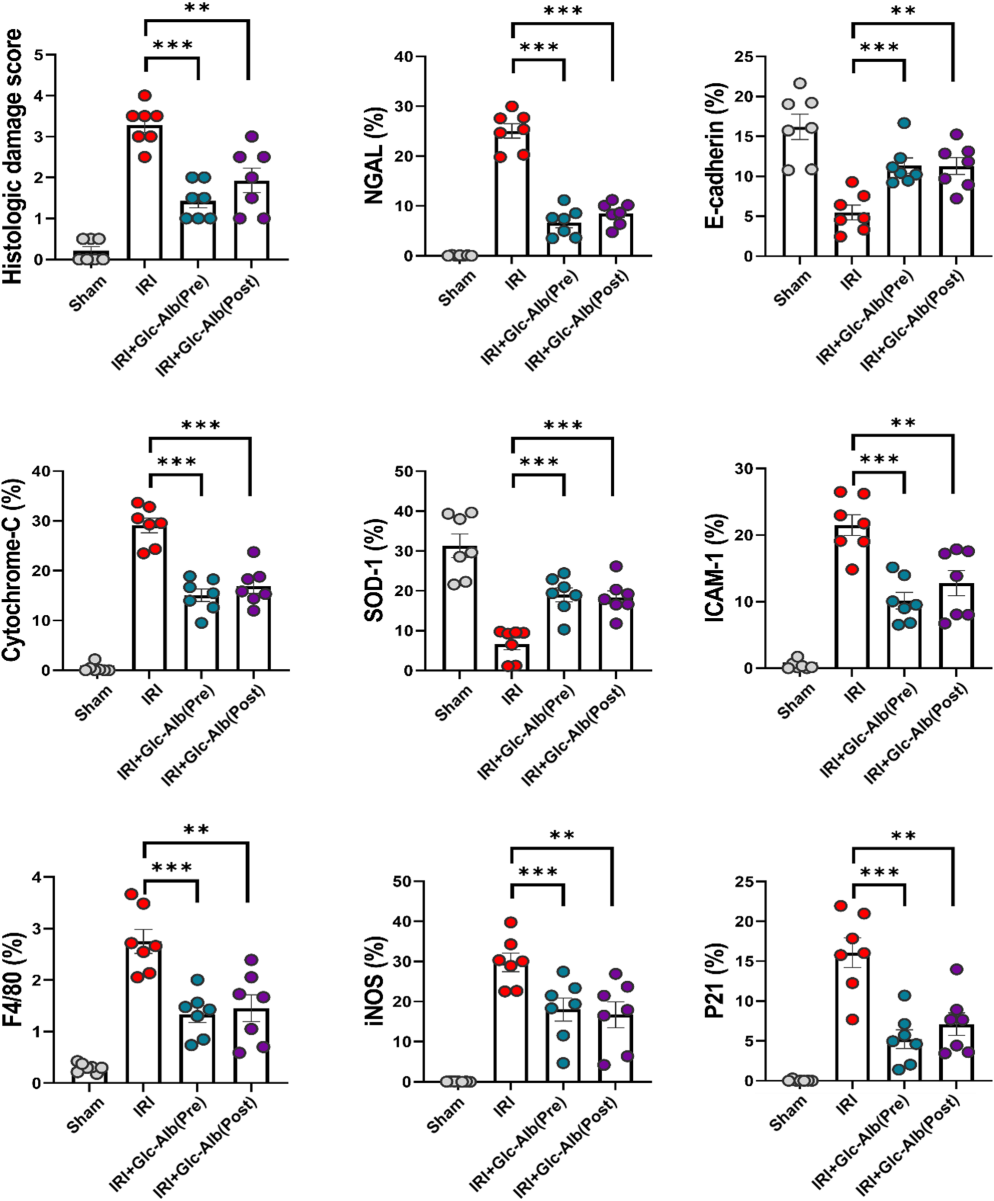
Improvement of renal injury following pre- and post-treatment of Glc^6^-AD^11^-Alb. **A.** Schematic illustration of the therapeutic mechanism of Glc^6^-AD^11^-Alb in inflammatory M1 macrophages, highlighting GLUT-mediated uptake, ROS reduction, suppression of inflammatory signaling, and preservation of mitochondrial and renal function. **B**. Changes in renal function following administration of Glc^6^-AD^11^-Alb in Sham, bIRI, pre-treatment, and post-treatment groups. **C.** Alterations in oxidative stress, inflammatory, apoptotic, and repair markers in renal tissue following administration of Glc^6^-AD^11^-Alb. Each graph quantitatively illustrates marker expression within the renal tissue. NGAL, neutrophil gelatinase-associated lipocalin; E-cadherin; Cytochrome C; SOD-1, superoxide dismutase; ICAM-1, intercellular adhesion molecule-1; F4/80; iNOS, inducible nitric oxide synthase; p21, cyclin-dependent kinase inhibitor

To further validate the therapeutic efficacy of Glc^6^-AD^11^-Alb in AKI, we examined its impact on renal function and molecular markers in pre- and post-treatment settings. The proposed mechanism is schematically illustrated in Fig. 4a, showing GLUT-mediated uptake of Glc^6^-AD^11^-Alb by inflammatory M1 macrophages, subsequent suppression of ROS generation, mitigation of inflammatory signaling cascades, and preservation of mitochondrial function, ultimately leading to improved renal function.

Building on the *in vivo* PET imaging data in Fig. 3, which confirmed preferential renal accumulation of Glc^6^-AD^11^-Alb in IRI-induced AKI models, we next investigated whether this targeted delivery translates into therapeutic benefits. We examined the effects of Glc^6^-AD^11^-Alb treatment on renal injury, dividing mice into four groups: Sham, bIRI, pre-treatment, and post-treatment. Pre- and post-treatment groups demonstrated markedly reduced serum BUN/Cr levels compared with the bIRI group, suggesting that Glc^6^-AD^11^-Alb effectively attenuates kidney injury even when administered after the ischemic insult. (Fig. 4b).

**Fig. 5:**
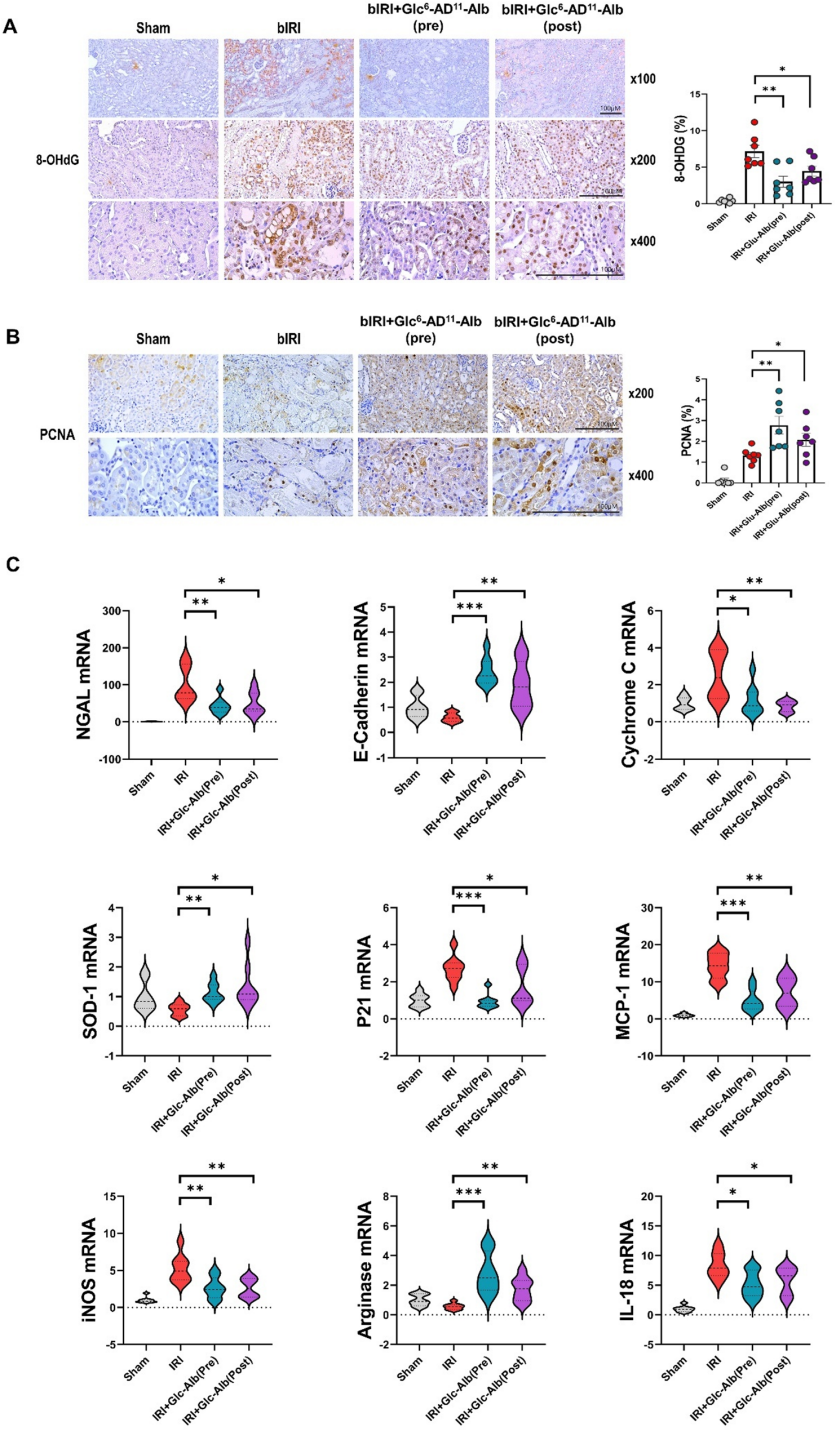
Improvement of renal injury following pre- and post-treatment of Glc^6^-AD^11^-Alb. **A-B.** Alterations in 8-OHdG and PCNA in renal tissue following administration of Glc^6^-AD^11^-Alb. Each graph illustrates marker expression within the renal tissue. **C.** Changes in mRNA expression levels in renal tissue following Glc^6^-AD^11^-Alb administration. NGAL, neutrophil gelatinase-associated lipocalin; E-cadherin; Cytochrome C; SOD-1, superoxide dismutase; ICAM-1, intercellular adhesion molecule-1; F4/80; iNOS, inducible nitric oxide synthase; p21, cyclin-dependent kinase inhibitor; 8-OHdG, 8-hydroxy-2’-deoxyguanosine; PCNA, proliferating cell nuclear antigen; MCP-1, monocyte chemoattractant protein-1; Arginase; IL-18, interleukin 18

Consistently, both treatment groups exhibited statistically significant reductions in histologic damage scores. Within renal tissue, Glc^6^-AD^11^-Alb treatment reduced the expression of injury and inflammatory markers, including NGAL, ICAM-1, F4/80, and iNOS, while simultaneously enhancing protective markers such as E-cadherin and SOD-1, regardless of treatment timing (Fig. 4c). Notably, levels of cytochrome C and p21, both associated with mitochondrial dysfunction and apoptosis, were significantly decreased, indicating that Glc^6^-AD^11^-Alb alleviates apoptosis and preserves mitochondrial integrity. The oxidative stress biomarker 8-OHdG, which was markedly elevated in the bIRI group, was also significantly reduced following treatment (Fig. 5a). Conversely, PCNA expression, a marker of proliferation and repair, was robustly increased, demonstrating enhanced regenerative capacity within renal tissue (Fig. 5b).

At the transcriptional level, changes in mRNA expression paralleled the immunohistochemistry results (Fig. 5c). Glc^6^-AD^11^-Alb administration reduced the expression of NGAL, MCP-1, iNOS, and p21, while upregulating E-cadherin, SOD-1, and arginase, the latter being implicated in macrophage-mediated tubular regeneration [30]. Importantly, IL-18, a pro-inflammatory cytokine linked to interferon-γ induction, was significantly decreased following treatment.

These findings align with previous reports demonstrating the central role of macrophage-driven inflammation and mtROS in the pathogenesis of IRI-induced AKI [6, 9], as well as the protective role of antioxidant mechanisms in maintaining tubular integrity [1]. Moreover, the observed upregulation of arginase is consistent with its reported role in tubular regeneration [30]. Collectively, these results highlight that Glc^6^-AD^11^-Alb exerts multifaceted protective effects in ischemia–reperfusion-induced AKI by modulating macrophage-driven inflammation, attenuating oxidative stress, preserving mitochondrial function, and enhancing tubular repair. Importantly, the comparable efficacy between pre- and post-treatment highlights the translational potential of this nanoplatform for clinical application, where therapeutic intervention is often initiated after the onset of injury.

### Anti-apoptotic effect and mitochondrial recovery by Glc^6^-AD^11^-Alb in an oxidative stress model

In the IRI–AKI setting, infiltrating macrophages and resident tubular epithelial cells contribute to disease progression through mtROS overproduction, albeit via distinct mechanisms: macrophages amplify inflammatory cascades through phagocytosis and inflammasome activation, whereas hPTECs undergo direct mitochondrial injury following ischemia/reperfusion stress [6, 9, 31]. Building on the PET imaging and *in vivo* findings, we next examined whether Glc^6^-AD^11^-Alb could exert direct protective effects on tubular cells, thereby complementing its macrophage-targeting function.

**Fig. 6:**
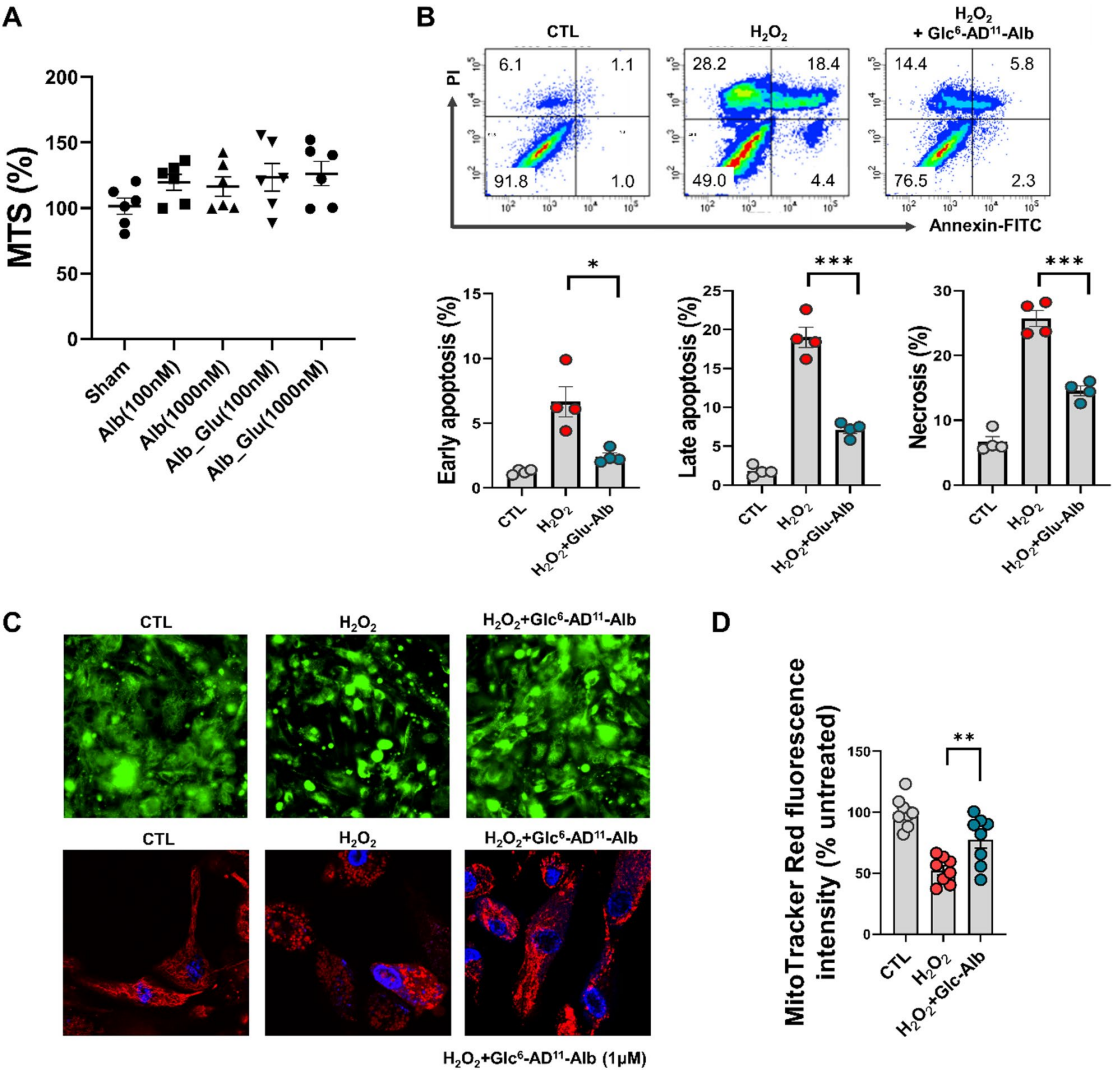
Anti-apoptotic effect and mitochondrial recovery by Glc^6^-AD^11^-Alb in an oxidative stress model. **(A)** MTS assays showed that Glc^6^-AD^11^-Alb exhibited no cytotoxicity even at the highest tested dose. **(B)** In the oxidative stress model using hPTECs treated with H₂O₂, Glc^6^-AD^11^-Alb reduced early and late apoptosis, as well as necrosis. **(C)** Changes in mitochondrial morphology and membrane potential assessed by confocal microscopy. **(D)** Glc^6^-AD^11^-Alb treatment restored mitochondrial morphology, preventing fragmentation and preserving network integrity under H₂O₂ stimulation. All samples were tested with Glc^6^-AD^11^-Alb at a concentration of 1µM

Cytotoxicity was first assessed across a range of concentrations, and MTS assays confirmed that Glc^6^-AD^11^-Alb exhibited no cytotoxicity even at the highest tested dose (Fig. 6a). Based on these findings, a working concentration of 1,000 nM (1 µM) was selected for subsequent experiments. In the oxidative stress model induced by H₂O₂, treatment with Glc^6^-AD^11^-Alb significantly reduced early and late apoptosis, as well as necrosis, in hPTECs (Fig. 6b). These results align with the proposed role of ROS scavenging in suppressing apoptotic signaling and maintaining mitochondrial homeostasis [32].

Mitochondrial morphology and function were further examined to elucidate the underlying mechanism. Under H₂O₂ stimulation, hPTECs displayed swollen, fragmented, or hyperfused mitochondria, accompanied by collapse of the mitochondrial network. In contrast, Glc^6^-AD^11^-Alb treatment restored mitochondrial morphology, preventing fragmentation and preserving network integrity (Fig. 6c, d). Together with reduced cytochrome C release and increased SOD-1 expression observed *in vivo*, these findings indicate that Glc^6^-AD^11^-Alb preserves mitochondrial integrity under oxidative stress.

Collectively, these results suggest that Glc^6^-AD^11^-Alb exerts dual therapeutic actions by suppressing macrophage-driven inflammatory signaling through GLUT-mediated uptake while simultaneously protecting hPTECs by mitigating ROS-induced mitochondrial dysfunction. This integrated mechanism provides a strong translational rationale for ROS-scavenging nanoplatforms as targeted therapeutics for AKI, as it addresses the inflammatory amplification caused by infiltrating macrophages [6] and the mitochondrial injury sustained by proximal tubular cells [9, 31]. By coordinating these effects across immune and parenchymal compartments, Glc^6^-AD^11^-Alb not only attenuates acute tissue damage but also preserves tubular integrity and reduces the risk of maladaptive repair, thereby lowering the likelihood of progression toward CKD [8, 10].

### Glucose-Alb rescues renal cells by restoring mitochondrial membrane potential under oxidative and hypoxic stress

To directly evaluate whether Glc^6^-AD^11^-Alb could restore mitochondrial homeostasis, we employed the JC-1 assay, a widely used method for assessing mitochondrial membrane potential. In this system, intact and polarized mitochondria emit a red fluorescent signal as a result of JC-1 aggregate formation, whereas damaged and depolarized mitochondria show green fluorescence from monomeric forms. Thus, the red-to-green ratio provides a direct readout of mitochondrial functional status in stressed renal epithelial cells.

We selected human primary tubular epithelial cells (hTECs) as the *in vitro* model instead of immortalized proximal tubular cell lines because primary cells better recapitulate the physiological and heterogeneous responses of renal tubular epithelia. Although proximal tubular cells represent the main site of ischemic damage, AKI pathophysiology involves multiple tubular segments. Importantly, primary hTECs retain native mitochondrial morphology, metabolic activity, and stress signaling pathways, thereby providing higher translational relevance for evaluating therapeutic effects on mitochondrial protection [8, 31].

**Fig. 7:**
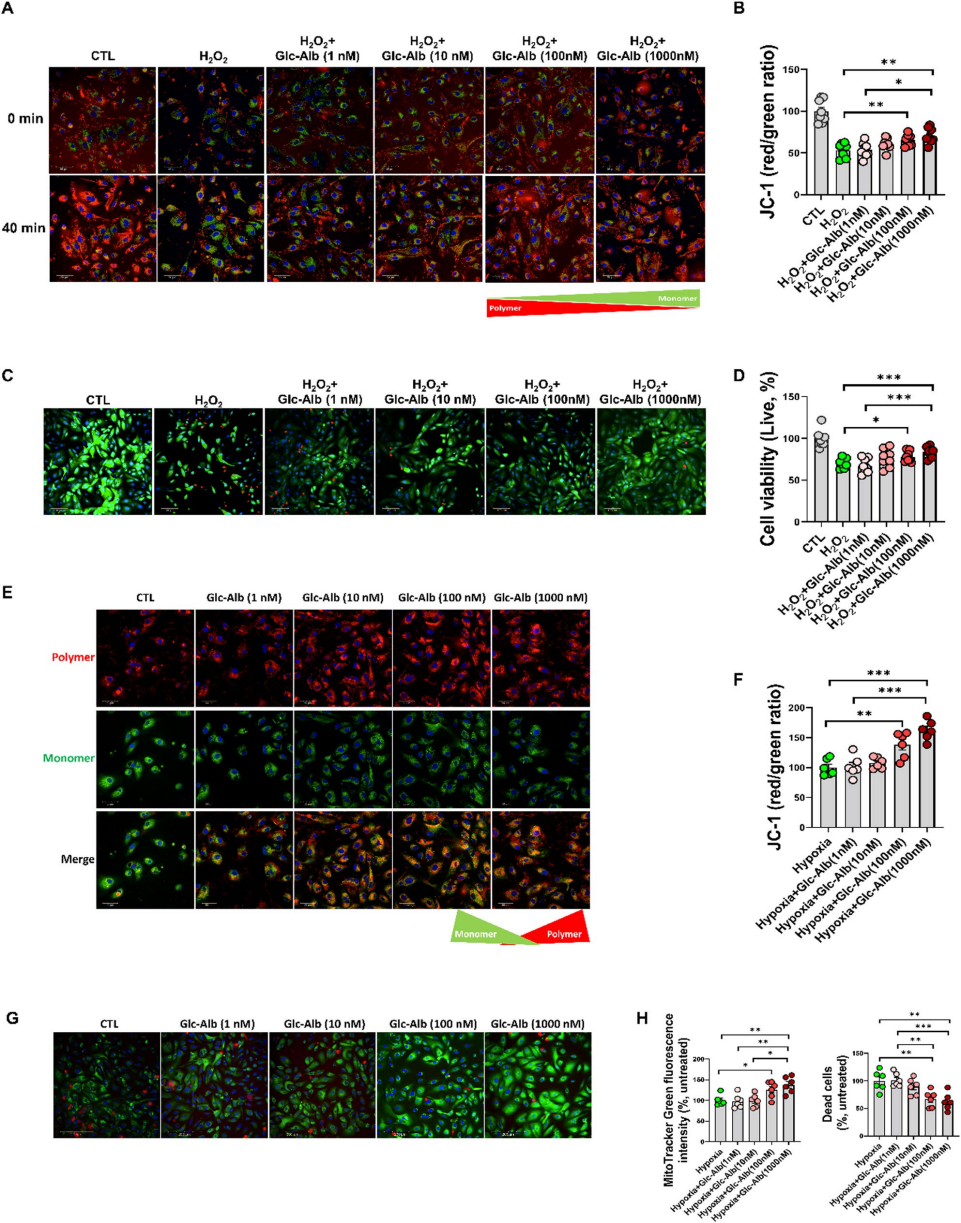
Glc^6^-AD^11^-Alb restores mitochondrial membrane potential and viability of human tubular epithelial cells under oxidative and hypoxic stress. **A** and **B.** Representative images and quantification of JC-1 under oxidative stress. hTECs were treated with H_2_O_2_ (1mM) and Glc^6^-AD^11^-Alb (10, 100, 1,000 nM). Green (monomer) and red (aggregate) JC-1 signals indicate mitochondrial depolarization and polarization, respectively. **C** and **D.** Cell viability was quantified and visualized after exposure to H_2_O_2_ (1 mM) and Glc^6^-AD^11^-Alb (10, 100, 1,000 nM) for 40 min. **E** and **F.** Images and quantification of hTECs after 24 h of hypoxic stress with Glc^6^-AD^11^-Alb treatment in a dose-dependent manner (10, 100, 1,000 nM). **G** and **H.** Mitotracker Green and EthD were visualized and quantified to assess mitochondrial membrane potential and cell survival in renal cells. Data are shown as mean ± standard error of the mean. *P < 0.05, **P < 0.01, ***P < 0.001

Our results revealed that Glc^6^-AD^11^-Alb treatment significantly preserved mitochondrial membrane potential in a dose-dependent manner. Under oxidative stress induced by H_2_O_2_, untreated cells showed a dominance of green fluorescence, indicating depolarization and collapse of mitochondrial integrity. By contrast, Glc^6^-AD^11^-Alb restored the red signal corresponding to polarized mitochondria, reflecting the recovery of mitochondrial function (Fig. 7a, b). In parallel, Glc^6^-AD^11^-Alb treatment improved overall cell viability and reduced EthD uptake, demonstrating that preservation of mitochondrial membrane potential translated into increased survival of tubular epithelial cells under stress conditions (Fig. 7c, d).

Hypoxic stress, which exacerbates ROS production and metabolic imbalance, further validated these findings. After 24 h of hypoxia, hTECs exhibited fragmented mitochondrial networks and compromised survival. Administration of Glc^6^-AD^11^-Alb markedly improved mitochondrial polarization, increased red JC-1 signal intensity, and sustained cell viability in a dose-dependent manner (Fig. 7e–h).

These results align with prior studies showing that mtROS disrupt mitochondrial DNA maintenance and drive epithelial injury in AKI [9], and they also resonate with more recent work demonstrating that nanoplatform-based antioxidant strategies can effectively sustain mitochondrial potential and prevent apoptosis during ischemia/reperfusion injury [12].

### Functional validation of mitochondrial recovery by Glc^6^-AD^11^-Alb under oxidative stress

Building on the evidence that Glc^6^-AD^11^-Alb preserved mitochondrial membrane potential and prevented apoptosis in hTECs (Fig. 7), we next evaluated whether these structural protections translated into functional recovery of mitochondrial respiration. Restoration of mitochondrial bioenergetics is a critical determinant of tubular cell survival in AKI, as oxidative stress not only disrupts mitochondrial membrane potential but also suppresses oxidative phosphorylation and promotes compensatory glycolysis [8, 9].

**Fig. 8:**
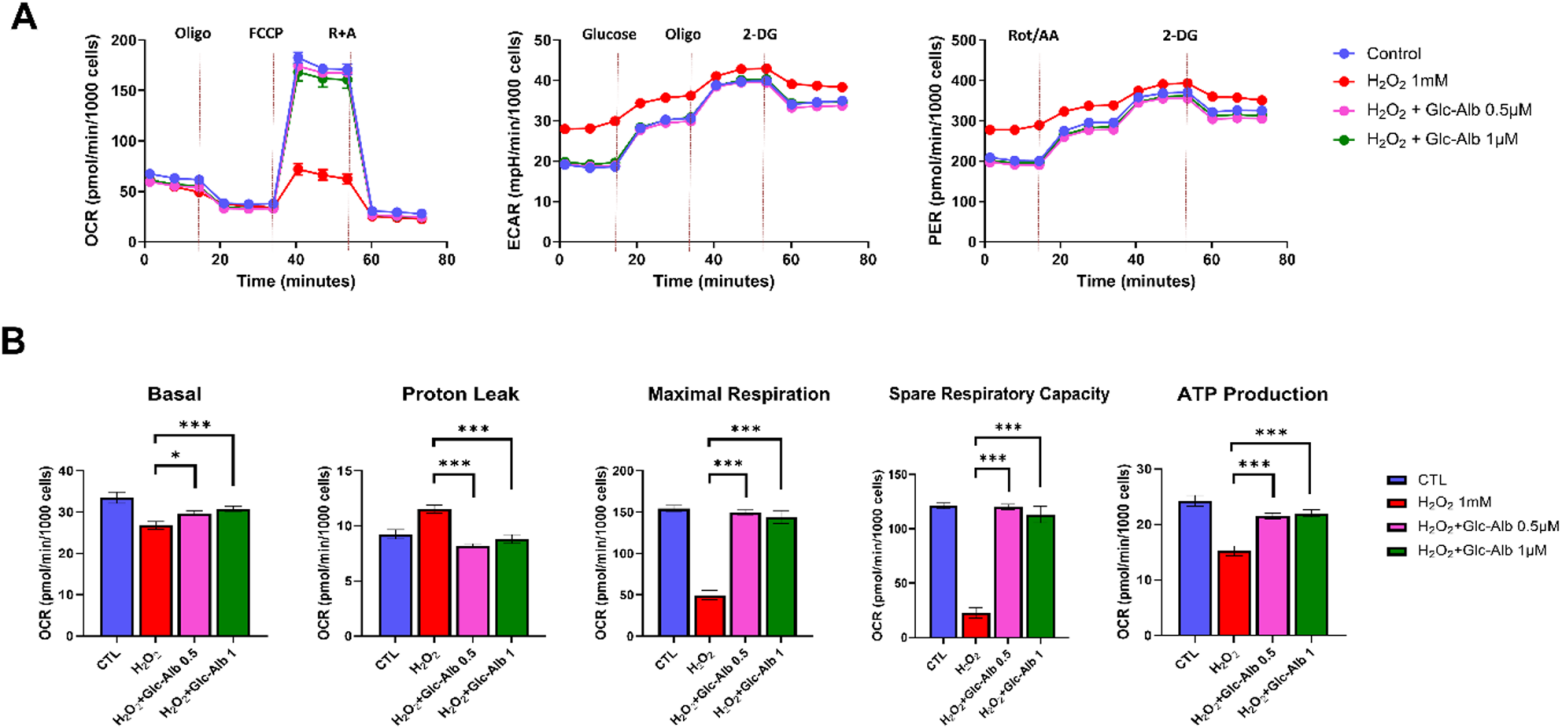
Functional validation of mitochondrial recovery by Glc^6^-AD^11^-Alb through Seahorse XF analysis. Glc^6^-AD^11^-Alb enhances mitochondrial respiration in renal cells under oxidative stress. **(A)** Representative Seahorse analysis plots of oxygen consumption rate (OCR), extracellular acidification rate (ECAR), and proton efflux rate (PER) in hTECs. Cells were treated with 1 mM H_2_O_2_ and Glc^6^-AD^11^-Alb at concentrations of 0.5 or 1 µM. Injections of oligomycin (2 µM), FCCP (1.5 µM), and rotenone/antimycin (0.5 µM) were used to assess various phases and parameters of mitochondrial respiration (*n* = 22). **(B)** Bar graphs display OCR values corresponding to basal respiration, proton leak, maximal respiration, spare respiratory capacity, and ATP production. Data are presented as mean ± standard error of the mean. *P < 0.05, **P < 0.01, ***P < 0.001

To address this, we employed the Seahorse XF Mito Stress Test, which provides a functional readout of energy metabolism by simultaneously measuring OCR and extracellular acidification rate (ECAR) [33]. In hTECs subjected to H₂O₂-induced oxidative stress, Glc^6^-AD^11^-Alb treatment markedly increased OCR while reducing ECAR and proton efflux rate (PER) (Fig. 8a), indicating enhanced oxidative phosphorylation and reduced glycolytic dependency. These findings suggest that Glc^6^-AD^11^-Alb not only mitigates mitochondrial dysfunction but also rebalances cellular metabolism away from glycolysis toward efficient respiration.

Quantitative analysis further confirmed significant increases in basal respiration, maximal respiration, ATP production, spare respiratory capacity, and coupling efficiency, whereas proton leak was reduced (Fig. 8b, S6). Reduced proton leak reflects more efficient coupling of electron transport to ATP synthesis, a critical feature for energy-demanding renal tubular cells (Fig. S7) [34]. Together, these data demonstrate that Glc^6^-AD^11^-Alb promotes mitochondrial ATP generation while suppressing maladaptive glycolysis, ultimately restoring cellular energy homeostasis under oxidative stress.

These results are consistent with prior work showing that ROS-driven mitochondrial dysfunction underlies AKI progression [8, 9], and that modulation of metabolic reprogramming is a promising therapeutic strategy [35]. Collectively, the Seahorse assay results functionally validate that Glc^6^-AD^11^-Alb restores mitochondrial respiration, complementing the membrane potential and anti-apoptotic effects demonstrated in Fig. 7.

### Mitochondrial ultrastructure preservation by Glc^6^-AD^11^-Alb

**Fig. 9:**
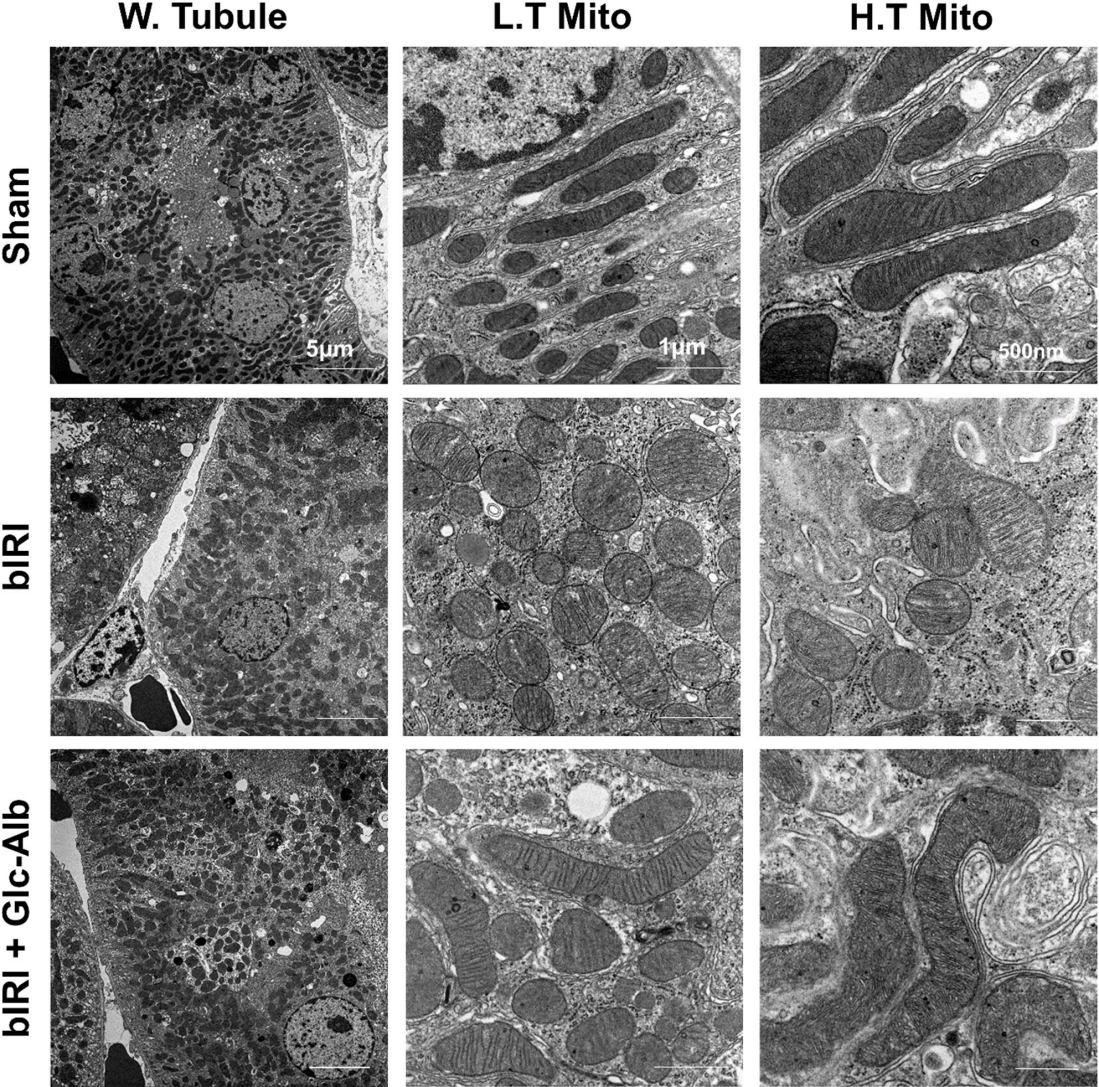
Glc^6^-AD^11^-Alb preserves mitochondrial ultrastructure in renal tissue following ischemia/reperfusion injury. Glc^6^-AD^11^-Alb reduced mitochondrial damage in the tubules of the bIRI mouse model. Representative TEM images of kidney sections show mitochondria in tubules (T. Mito). Scale bars: 5 μm (×5,000; W. Tubule), 1 μm (×30,000; L. T. Mito), and 500 nm (×50,000; H. T. Mito). (W. Tubule = Whole Tubule, L. T. Mito = Lower-magnification TEM image of mitochondria in tubules, H.T. Mito = Higher-magnification TEM image of mitochondria in tubules)

Transmission electron microscopy (TEM) was employed to provide morphological validation of the protective effects of Glc^6^-AD^11^-Alb on renal mitochondria. In the bIRI group, mitochondria exhibited characteristic signs of IRI, including swelling, cristae loss, and vacuolated matrices. By contrast, Glc^6^-AD^11^-Alb treatment preserved mitochondrial ultrastructure, as evidenced by elongated morphology, intact and densely packed cristae, and uniformly electron-dense matrices (Fig. 9).

These observations are consistent with previous reports that ischemia-induced mtROS disrupt cristae structure and impair ATP synthesis [8, 9], and that nanoplatform-based antioxidant interventions can restore mitochondrial ultrastructure in AKI [12]. Taken together with the functional findings from JC-1 and Seahorse assays (Figs. 7 and 8), the TEM results corroborate that Glc^6^-AD^11^-Alb maintains mitochondrial homeostasis at structural and functional levels, thereby supporting its therapeutic potential in ischemia/reperfusion-induced AKI.

## Conclusions

We developed a glucosylated Alb nanoplatform (Glc^6^-AD^11^-Alb) that targets inflammatory macrophages and tubular epithelial cells, thereby alleviating oxidative stress and preserving mitochondrial function in ischemia–reperfusion-induced AKI. Glc^6^-AD^11^-Alb reduced ROS levels, suppressed apoptosis, and restored mitochondrial membrane potential *in vitro*, whereas Seahorse assays confirmed recovery of oxidative phosphorylation with reduced glycolytic dependency. *In vivo*, it selectively accumulated in injured kidneys, improved renal function, attenuated tissue damage, and promoted tubular regeneration. TEM further demonstrated preservation of cristae structure and mitochondrial morphology. Although the precise balance between extracellular and intracellular actions warrants further investigation, the injury-selective accumulation and multivalent structure of Glc^6^-AD^11^-Alb provide a mechanistic basis for mitochondrial protection that cannot be achieved by endogenous serum albumin alone.

Overall, these findings show that Glc^6^-AD^11^-Alb confers therapeutic benefits by simultaneously modulating immune responses and protecting mitochondrial integrity, establishing it as a promising translational strategy for AKI. Our results align with recent advances in nanomedicine using antioxidant platforms to restore mitochondrial function [36, 37], highlighting the potential of Alb-based nanoplatforms as precision therapeutics for oxidative stress-related diseases.

## Supplementary Information

Supplementary Material

## Data Availability

No datasets were generated or analysed during the current study.

## Abbreviations

ADIBO: Azadibenzocyclooctyne
Alb: Albumin
AKI: Acute kidney injury
bIRI: Bilateral ischemia–reperfusion injury
BUN: Blood urea nitrogen
CKD: Chronic kidney disease
Cr: Creatinine
DAPI: 4’,6-diamidino-2-phenylindole
DLS: Dynamic light scattering
DMSO: Dimethyl sulfoxide
DOF: Degree of functionalization
EM: Electron microscopy
ECAR: Extracellular acidification rate
EthD: Ethidium homodimer
FITC: Fluorescein isothiocyanate
FNR648: a zido-Flamma 648
GAPDH: Glyceraldehyde-3-phosphate dehydrogenase
Glc^6^-AD^11^-Alb: Glucosylated albumin with 6 glucose and 11 ADIBO moieties
GLUT1: Glucose transporter 1
hPTECs: Human proximal tubular epithelial cells
hTECs: Human primary tubular epithelial cells
H_2_O_2_: Hydrogen peroxide
IFN-γ: Interferon-gamma
IRI: Ischemia–reperfusion injury
ITLC-SG: Instant thin-layer chromatography silica gel
JC-1: 5,5′,6,6′-Tetrachloro-1,1′,3,3′-tetraethylbenzimidazolylcarbocyanine iodide
LPS: Lipopolysaccharide
MALDI-TOF: Matrix-assisted laser desorption/ionization time-of-flight
mPTP: Mitochondrial permeability transition pore
mtROS: Mitochondrial reactive oxygen species
NGAL: Neutrophil gelatinase-associated lipocalin
NOTA: 1,4,7-Triazacyclononane-1,4,7-triacetic acid
OCR: Oxygen consumption rate
PBS: Phosphate-buffered saline
PCNA: Proliferating cell nuclear antigen
PER: Proton efflux rate
PET: Positron emission tomography
PI: Propidium iodide
PMS: Phenazine methosulfate
PTEC: Proximal tubular epithelial cells
qRT-PCR: Quantitative reverse transcription polymerase chain reaction
rHSA: Recombinant human serum albumin
ROS: Reactive oxygen species
RNS: Reactive nitrogen species
SOD-1: Superoxide dismutase 1
SPAAC: Strain-promoted azide–alkyne cycloaddition
TEM: Transmission electron microscopy
TLC: Thin-layer chromatography
uIRI: Unilateral ischemia-reperfusion injury
