# Supplementary material for: Optimization of M1 macrophage targeting using a glucosylated albumin nanoplatform for ROS scavenging and mitochondrial rescue in acute kidney injury
Source: J Nanobiotechnology. 2026 Jan 29;24:187. doi: 10.1186/s12951-026-04061-6 (PMC12924581; doi:10.1186/s12951-026-04061-6)
Supplement: Supplementary file 1 — Supplementary Material [file 12951_2026_4061_MOESM2_ESM.docx]

Supporting Information

**Optimization of M1 Macrophage Targeting Using a Glucosylated Albumin Nanoplatform for ROS Scavenging and Mitochondrial Rescue in Acute Kidney Injury**

Ji Yong Park^1,2,3,4†^, Jung Nam An^5†^, Seong Min Lee^9^, Young Chan Ann^1^, Eunjin Bae^6^, Kyung Don Yoo^7^, Yong Chul Kim^8^, Hyeri Chae^3,13^, Joo Yeon Oh^10^, Ran Ji Yoo^2^, Dong Ki Kim^8^, Sung Hyun Hong^14^, Yon Su Kim^8*^, Yun-Sang Lee^,1,2,3,4,12^*, and Seung Hee Yang^10,11^*

^1^Institute of Radiation Medicine, Seoul National University Medical Research Center, Republic of Korea

^2^Department of Nuclear Medicine, Seoul National University Hospital, Republic of Korea

^3^Department of Nuclear Medicine, Seoul National University College of Medicine, Republic of Korea

^4^Cancer Research Institute, Seoul National University, Republic of Korea

^5^Department of Internal Medicine, Hallym University Sacred Heart Hospital, Republic of Korea

^6^Department of Internal Medicine, Gyeongsang National University Hospital, Republic of Korea

^7^Department of Internal Medicine, University of Ulsan College of Medicine, Republic of Korea

^8^Department of Internal Medicine, Seoul National University Hospital, Republic of Korea

^9^Department of Biomedical Sciences, College of Medicine, Seoul National University, Republic of Korea

^10^Biomedical Research Institute, Seoul National University Hospital, Republic of Korea

^11^Seoul National University Kidney Research Institute, Republic of Korea

^12^Research Institute for Convergence Science, Seoul National University, Republic of Korea ^13^Department of Molecular Medicine and Biopharmaceutical Sciences, Graduate School of Convergence Science and Technology, Seoul National University, Republic of Korea

^14^Clichembio Inc, Republic of Korea

***Correspondence**

Seung Hee Yang, PhD

Professor

Biomedical Research Institute, Seoul National University Hospital, Seoul, South Korea

Department of Kidney Research Institute, Seoul National University Medical Research Center, Seoul, South Korea

E-mail: [ysh5794@snu.ac.kr](mailto:ysh5794@snu.ac.kr)

Yun-Sang Lee, PhD

Professor

Department of Nuclear Medicine, Seoul National University College of Medicine, Seoul, Republic of Korea

E-mail: [wonza43@snu.ac.kr](mailto:wonza43@snu.ac.kr)

Yon Su Kim, MD.PhD

Professor

Department of Internal Medicine, Seoul National University Hospital, Seoul, Republic of Korea

E-mail: [yonsukim@snu.ac.kr](mailto:yonsukim@snu.ac.kr)

† These authors contributed equally to this work

**1. Figures**

**Figure S1. WST-1–based measurement of superoxide scavenging activity of Alb and Glc-Alb.** Alb and Glc-Alb were incubated with xanthine/xanthine oxidase–generated superoxide at increasing concentrations (0.25–4 µM), and the remaining O₂⁻ levels were quantified using the WST-1 assay according to the manufacturer’s instructions. Both Alb and Glc-Alb exhibited comparable dose-dependent superoxide scavenging activity, reaching saturation at approximately 4 µM, indicating that glucose modification does not alter the intrinsic antioxidant capacity of albumin. Data represent mean scavenging rate (%) relative to untreated control.


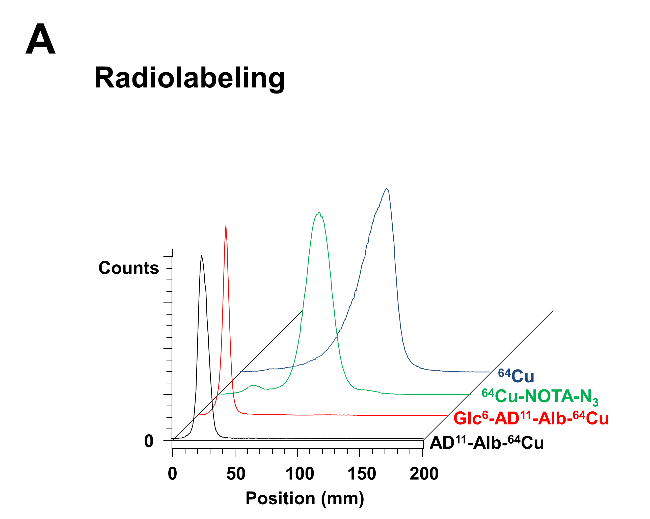

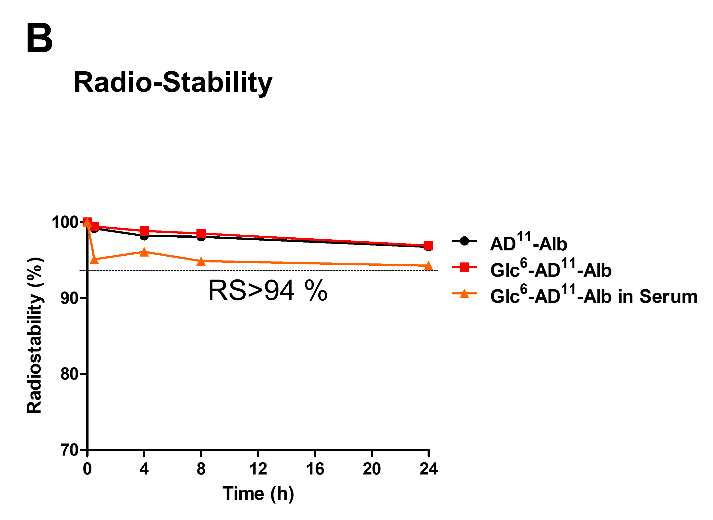


**Figure S2. Radiolabeling and radiostability of albumin nanoplatforms.** **A.** Radio-TLC chromatograms showing distinct Rf values of free ⁶⁴Cu (Rf = 0.8–1), [⁶⁴Cu]Cu-NOTA-N₃ (Rf = 0.5–0.6), AD^11^-Alb-⁶⁴Cu, and Glc^6^-AD^11^-Alb-⁶⁴Cu (Rf = 0–0.2), confirming successful radiolabeling with >99% efficiency.

**B.** Time-dependent radiostability of AD^11^-Alb-⁶⁴Cu and Glc^6^-AD^11^-Alb-⁶⁴Cu in PBS and serum. Both formulations exhibited excellent stability, maintaining >94% radiochemical purity for 24 h


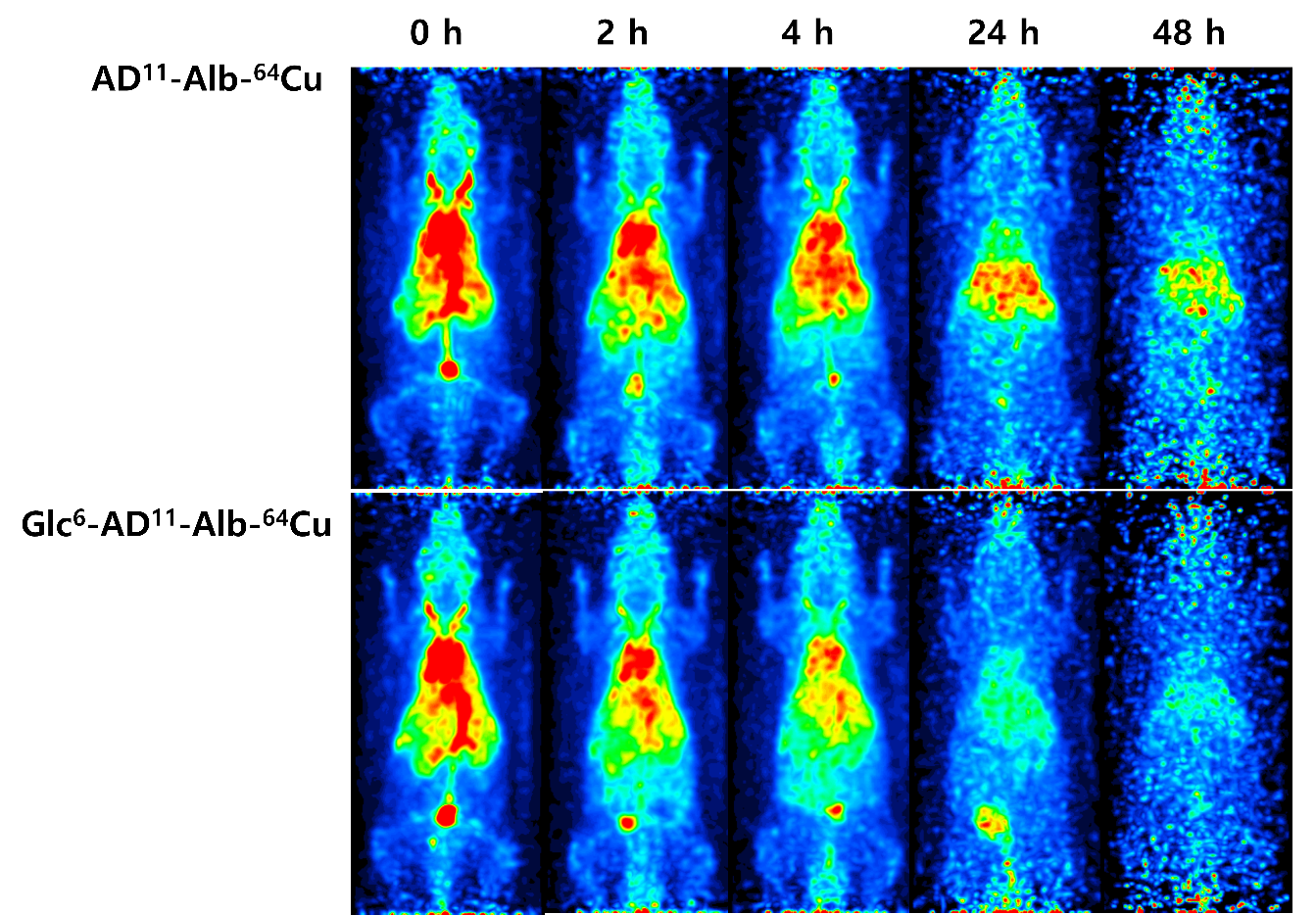


**Figure S3. *In vivo* PET imaging of AD^11^-Alb-⁶⁴Cu and Glc^6^-AD^11^-Alb-⁶⁴Cu in healthy mice.** Representative PET images acquired at 0, 2, 4, 24, and 48 h after intravenous injection of AD^11^-Alb-⁶⁴Cu (upper panel) or Glc^6^-AD^11^-Alb-⁶⁴Cu (lower panel). Both nanoplatforms showed prolonged circulation and predominant hepatic uptake. AD^11^-Alb-⁶⁴Cu exhibited sustained liver retention up to 24 h, whereas Glc^6^-AD^11^-Alb-⁶⁴Cu underwent early intestinal clearance starting at 4 h, likely resulting from hepatocyte-mediated uptake and biliary excretion. No significant renal uptake was detected for either formulation, consistent with the known pharmacokinetics of albumin-based carriers. Quantitative circulation half-lives (T1/2α and T1/2β) derived from image fitting are summarized in Table S2.


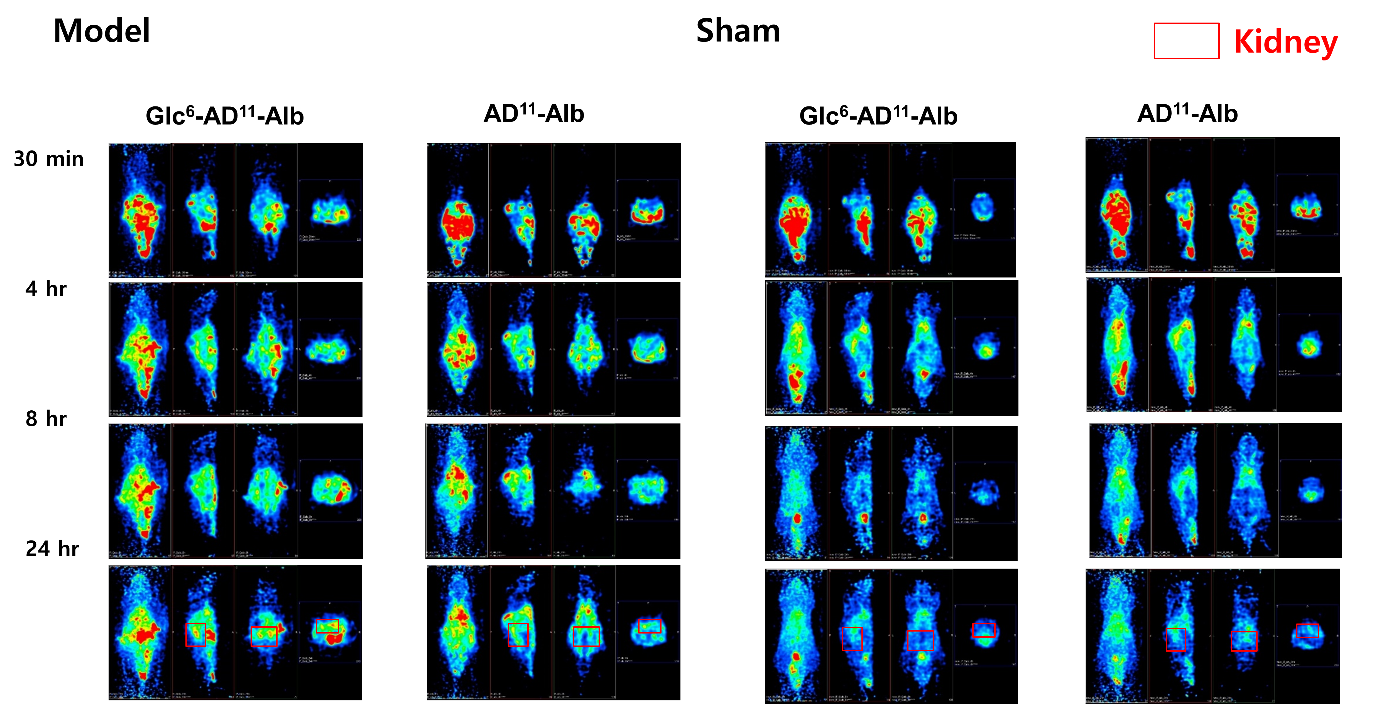


**Figure S4. *In vivo* PET imaging of AD¹¹-Alb-⁶⁴Cu and Glc⁶-AD¹¹-Alb-⁶⁴Cu in bilateral ischemia–reperfusion injury (bIRI) and Sham-operated mice.** Representative longitudinal PET images were acquired at 0.5, 4, 8, and 24 h post-injection. In the bIRI model, Glc⁶-AD¹¹-Alb-⁶⁴Cu demonstrated pronounced renal uptake and retention relative to AD¹¹-Alb-⁶⁴Cu, consistent with preferential targeting of inflamed kidneys. By contrast, Sham-operated mice primarily exhibited systemic circulation with negligible renal accumulation. The red box highlights the anatomical location of the kidneys in the images.


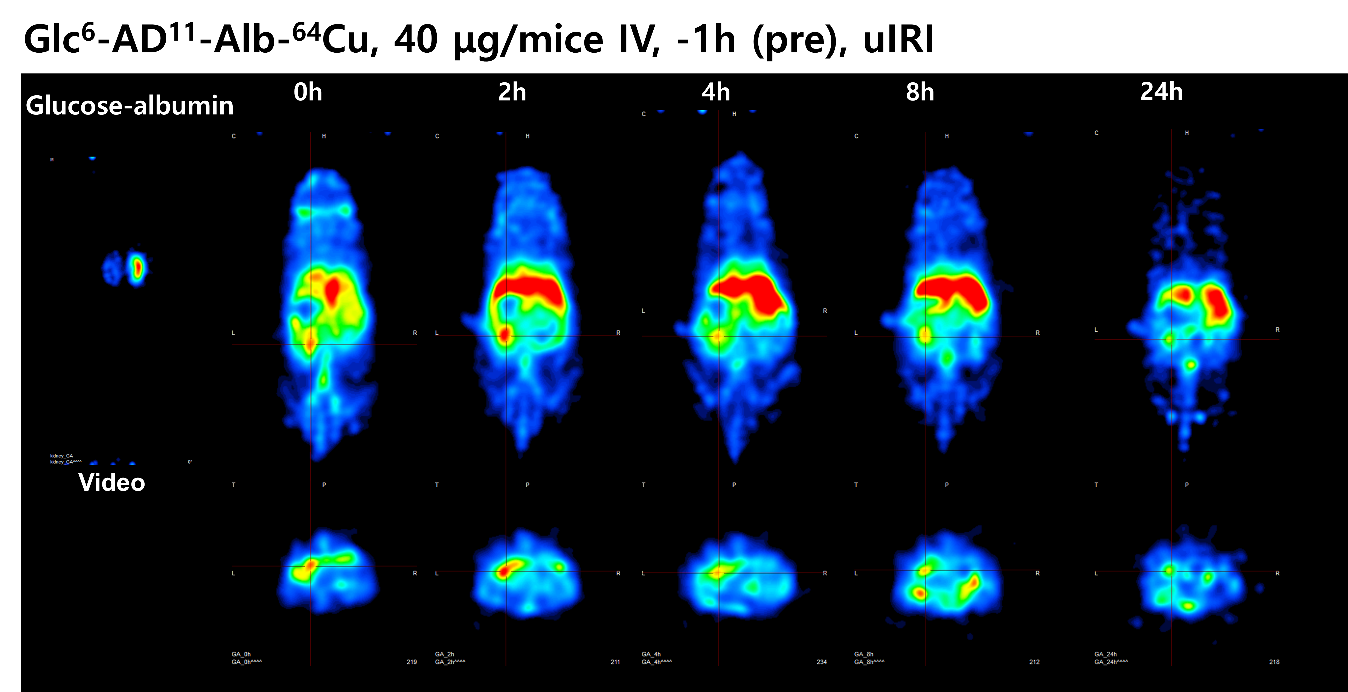


**Figure S5. *In vivo* PET imaging of Glc⁶-AD¹¹-Alb-⁶⁴Cu in a unilateral ischemia–reperfusion injury (uIRI) model.** Representative PET images acquired at 0, 2, 4, 8, and 24 h following intravenous injection of Glc⁶-AD¹¹-Alb-⁶⁴Cu (40 μg/mouse). Sectional analyses revealed negligible uptake in the contralateral normal kidney, whereas the IRI-affected kidney showed strong and persistent signals, particularly in coronal and transverse views. *Ex vivo* imaging of dissected kidneys further confirmed preferential accumulation of Glc⁶-AD¹¹-Alb-⁶⁴Cu in the injured renal parenchyma, highlighting its capability to selectively target early inflammatory environments *in vivo*.


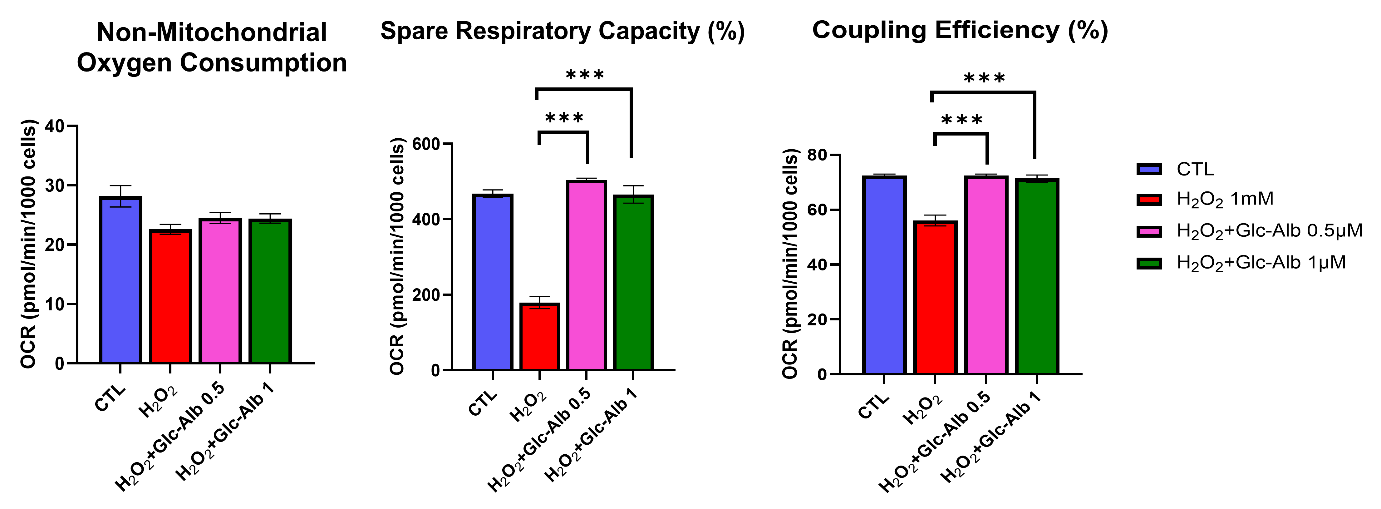


**Figure S6. Glucose-albumin enhances mitochondrial respiration and ATP production.** Representative Seahorse analysis plots for non-mitochondrial oxygen consumption, spare respiratory capacity (%), and coupling efficiency (%) are shown. Data are presented as mean ± SEM. *P < 0.05, **P < 0.01, ***P < 0.001.

**
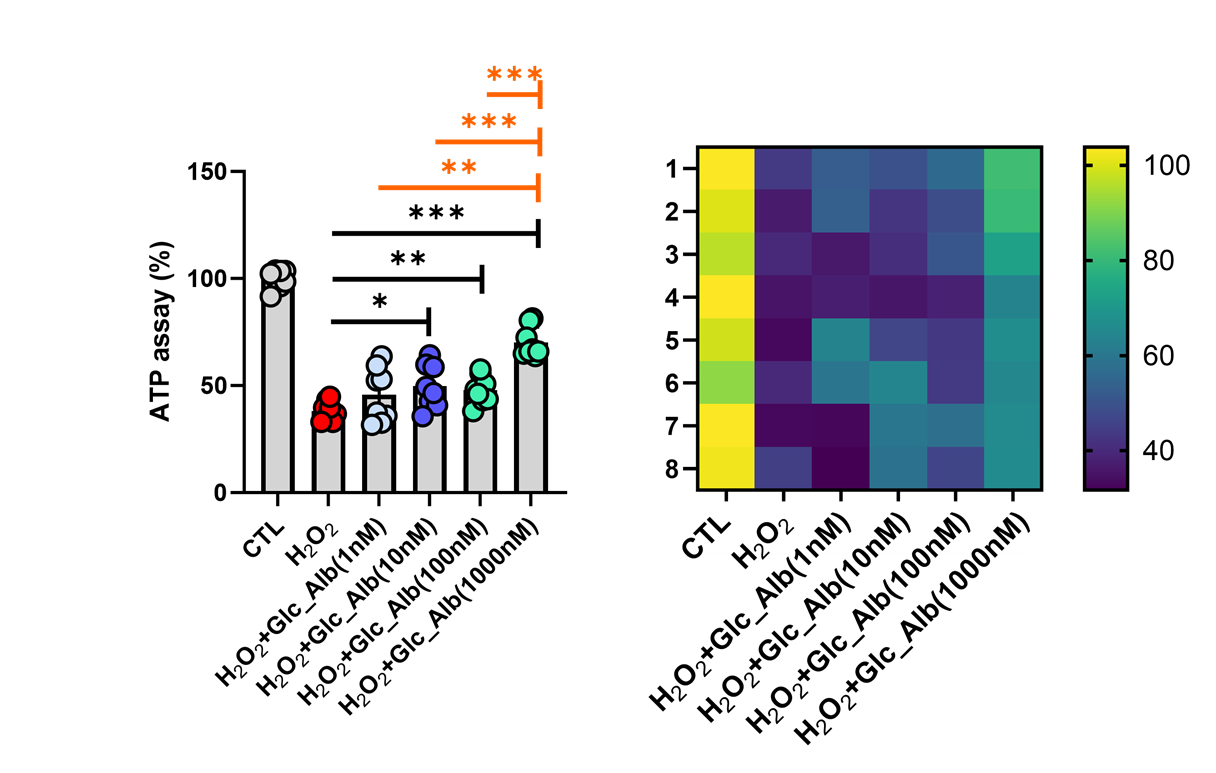
**

**Figure S7. Restoration of cellular ATP levels in oxidatively stressed tubular epithelial cells by Glc-Alb treatment.** Cellular ATP levels were quantified using a luminescence-based ATP detection assay. Primary human tubular epithelial cells were exposed to oxidative stress and treated with Alb or Glc⁶-AD¹¹-Alb as indicated. Luminescence signals, proportional to intracellular ATP content, were measured using a microplate luminometer. Glc⁶-AD¹¹-Alb treatment significantly restored ATP levels compared with oxidatively stressed controls, indicating preservation of mitochondrial energy metabolism. Data are presented as mean ± SEM. *P < 0.05, **P < 0.01, ***P < 0.001.

**2. Tables**


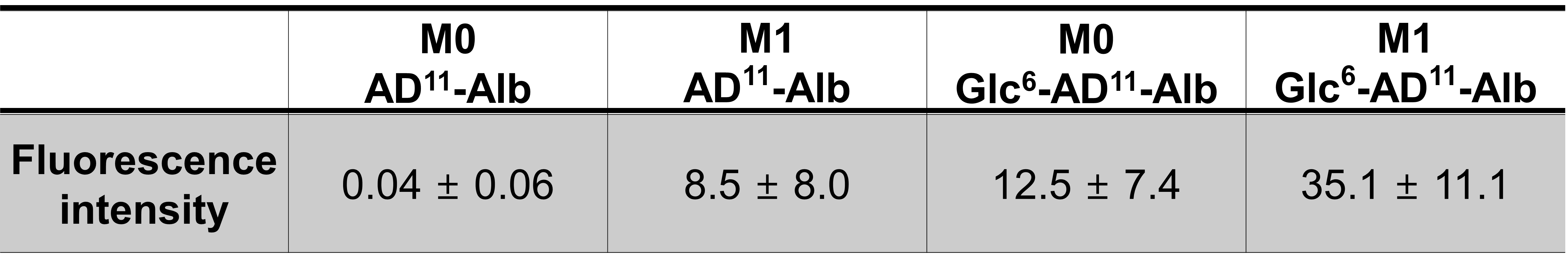


**Table S1. Fluorescence values from confocal imaging.** Mean Fluorescence Intensity (MFI) of signals in confocal images of M0 and M1 macrophages treated with AD^11^-Alb-FL or Glc^6^-AD^11^-Alb-FL. Data are expressed as mean ± SEM from three independent experiments. (**P < 0.01, ***P < 0.001).


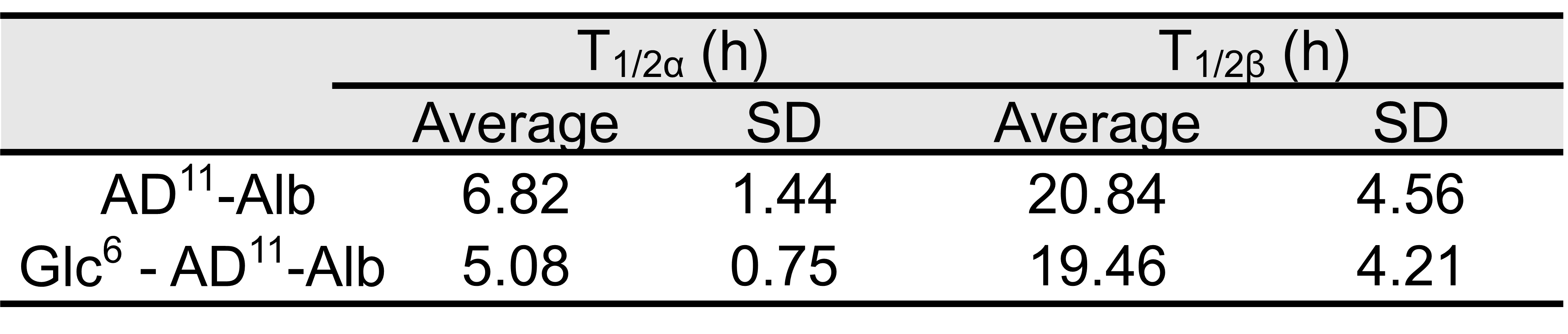


**Table S2. Pharmacokinetic parameters of albumin nanoplatforms.** Quantitative pharmacokinetic data of AD^11^-Alb-⁶⁴Cu and Glc^6^-AD^11^-Alb-⁶⁴Cu derived from PET imaging (Figure S2). Values were obtained by fitting time–activity curves from ROI analysis and are presented as mean ± SD (n = 4). Parameters include distribution half-life (T₁/₂α) and elimination half-life (T₁/₂β).
